# Maternal, Infant, Reproductive and Child Health in Cystic Fibrosis (MATRIARCH_CF): a prospective, observational study to evaluate pregnancy and parenthood in females with cystic fibrosis and health of the offspring in the CFTR-modulator era

**DOI:** 10.1136/bmjresp-2026-004270

**Published:** 2026-06-30

**Authors:** Amy Downes, Idan Bokobza, Ladina Weitnauer, Rebecca Scott, Rebecca Dobra, Christopher Short, Thomas Semple, Olivia Beaumont, Catherine Williamson, Jane C Davies, Imogen Felton

**Affiliations:** 1 Imperial College London Institute of Reproductive and Developmental Biology, London, UK; 2 Royal Brompton Hospital, Guy's and St Thomas’ NHS Foundation Trust, London, UK; 3 Imperial College London National Heart and Lung Institute, London, UK; 4 Chelsea and Westminster Hospital, London, UK; 5 King’s College Hospital NHS Foundation Trust, London, UK

**Keywords:** Cystic Fibrosis, Bronchiectasis, Imaging/CT MRI etc, Surveys and Questionnaires

## Abstract

**Introduction:**

Cystic fibrosis transmembrane conductance regulator (CFTR) modulators (CFTRm) have altered the landscape of pregnancy and parenthood in cystic fibrosis (CF). As pregnant and breastfeeding females with CF (FwCF) were excluded from pivotal CFTRm trials, evidence to guide management and counsel families on maternal and child outcomes is limited. While CFTRm are unlicensed for use in pregnancy, substantial clinical benefit means most continue therapy. Early studies indicate that CFTRm cross the placenta and are detectable in breastmilk; reported associations include infant liver dysfunction and cataracts but risks remain poorly characterised. We describe the protocol for the first UK-based prospective study evaluating pregnancy and parenthood in FwCF and health outcomes in their children in the CFTRm era.

**Methods and analysis:**

A single protocol underpins three linked prospective observational substudies in the MATRIARCH_CF research programme. The ‘Mama’ cohort recruits FwCF planning pregnancy or pregnant, with follow-up from preconception to 24 months postpartum, assessing physical and psychological health, lung function and CF-related complications. ‘Mini’ recruits children aged 0–24 months born to a parent with CF and compares offspring exposed and unexposed to CFTRm in utero and/or through breastfeeding; measures include birth outcomes, congenital anomalies, liver biochemistry, growth and neurodevelopment. ‘Midi’ recruits children aged 3–6 years from the same exposure groups and assesses neurodevelopment and lung function. Longitudinal data across cohorts will strengthen the evidence base, support risk–benefit discussions and inform clinical guidance.

**Ethics and dissemination:**

The protocol was approved by NHS ethics HSC REC A committee (25/NI/0027 19 March 2025). Assessments align with routine care where possible. Visit windows are flexible and participants may decline individual assessments. The research team does not influence CFTRm initiation, cessation or dose adjustment. People with CF contributed throughout study design to ensure acceptability. Findings will be disseminated through PhD theses, conference presentations and peer-reviewed publications.

**Trial registration number:**

NCT06797206.

**WHAT IS ALREADY KNOWN ON THIS TOPIC**

- Most studies examining pregnancy in females with cystic fibrosis (FwCF) predate the widespread use of disease-modifying cystic fibrosis transmembrane conductance regulator modulators (CFTRm) for people with CF (PwCF). Historical data suggest that FwCF are more likely than the general population to have poorer obstetric and fetal outcomes, including increased rates of preterm birth, gestational diabetes and congenital malformations. Recent studies suggest that these high rates of preterm birth and gestational diabetes persist despite CFTRm use.

**WHAT THIS STUDY ADDS**

- This is the first UK-based prospective study of CF pregnancy in the CFTRm era. It encompasses not only the maternal experience including obstetric and mental health outcomes but also neonatal and childhood outcomes in CFTRm-exposed offspring.
- Codesigned with expert-patient partners, the study addresses research priorities cited by PwCF to better understand their individualised risks relating to pregnancy and facilitate medication choices in pregnancy and breastfeeding in the modern CF-therapeutic era.

**HOW THIS STUDY MIGHT AFFECT RESEARCH, PUBLICATION OR POLICY**

- This study will provide prospective evidence in the rapidly evolving field of cystic fibrosis (CF)-maternal fetal medicine, contributing to the evidence base for improved individualised preconception counselling and pregnancy management for females with CF (FwCF) in the cystic fibrosis transmembrane conductance regulator modulators (CFTRm) era within a UK-based healthcare system.
- Prospective follow-up of offspring will provide data on potential short-term and long-term outcomes following in utero and breastmilk exposure to CFTRm. This data will be shared through peer-reviewed publications, informing the development of clinical advice documents for the management of pregnancy in FwCF, as well as the clinical follow-up of these offspring.

## Introduction

 MATRIARCH_CF is the first UK-based prospective observational study examining the impact of pregnancy on the health of females with cystic fibrosis (FwCF) in the cystic fibrosis transmembrane conductance regulator (CFTR) modulator era. It will characterise maternal experiences and obstetric outcomes of FwCF, and short-term and long-term health and neurodevelopmental outcomes of their children. The full study protocol is available in the online supplemental material.

**Figure 1:**
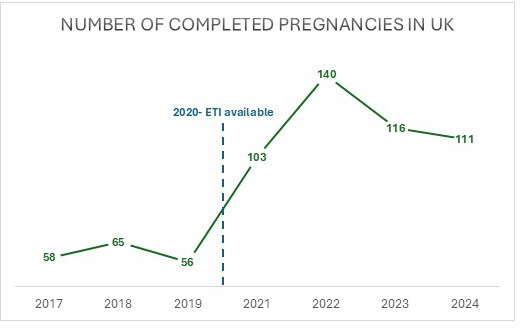
Number of completed pregnancies in FwCF (not including termination and miscarriage data) in the UK as taken from the CF Trust registry.^5^ CF, cystic fibrosis; FwCF, females with cystic fibrosis.

CFTR modulator (CFTRm) therapy, particularly elexacaftor/tezacaftor/ivacaftor (ETI), has led to rapid, life-changing improvements for many eligible people with CF (PwCF).^1^ Under current prescribing eligibility criteria, over 95% of PwCF in the UK or USA have at least one pathogenic variant which is considered to be CFTRm responsive and are thus able to benefit from these therapies, which can be prescribed from the age of 2 years (from 1 month of age for ivacaftor alone).^2^,^4^ CFTRm improve both lung function and nutritional status, both prognostic markers associated with CF survival and quality of life measures. This is reflected in advancing predicted survival age, now over 60 years.^15^,^7^ These advances have also positively impacted reproductive health in FwCF.^8 9^ This is demonstrated in the substantial and sustained increase in reported pregnancies among FwCF in those countries with widespread availability of CFTRm, including the UK, where ETI was first available in 2020 (figure 1).^5 10^ However, as a result of the rapid pace of change, clinical practice currently lacks up-to-date research and real-world evidence to guide counselling and optimal support for shared decision making between PwCF and healthcare professionals (HCPs) at all stages of the reproductive pathway from preconception counselling through pregnancy and into the post-partum phase.

The overarching study design described here aims to capture clinical outcomes and longitudinal health trajectories in the unique dyad of FwCF and their children, at a critical time for the use of novel therapies in pregnancy. It will also explore the experiences and considerations of FwCF through this important life-stage in the context of improving CF-prognosis and expanding life opportunities for many PwCF. As a result, shared decision making between prospective parents with CF and clinicians from a wide network of both CF and non-CF professionals will be better informed to receive and deliver up-to-date, individualised care in the modern CF therapeutic era.

To date, evidence of pregnancy outcomes in FwCF has largely been derived from retrospective studies, with small sample sizes, conducted in the pre–CFTRm era. Historically, FwCF experienced poorer obstetric outcomes than the general population, including higher rates of preterm birth, gestational diabetes and congenital anomalies.^11^,^13^ Those studies that have been published looking at data in more recent years show that thus far rates of preterm birth and gestational diabetes remain elevated, including in fwCF taking CFTRm throughout pregnancy.^14 15^

Notably, as in most interventional drug trial designs, pregnant individuals and those attempting to conceive have been excluded from CFTRm clinical trials, and these therapies remain unlicensed for use in pregnancy. As a result, evidence guiding CFTRm use during pregnancy and lactation is limited to case reports and retrospective studies.

In vivo animal data demonstrate that CFTRm compounds, including elexacaftor, tezacaftor and ivacaftor comprising the highly effective triple modulator Kaftrio(/ETI), cross the placenta, resulting in fetal exposure, with measurable drug levels also detected in breastmilk.^16^,^19^ Although available animal reproductive toxicology and clinical data have not identified a clear association with teratogenicity or congenital anomalies,^15^ currently there are limited clinical and preclinical reports of potential adverse effects on fetal development. These include cataracts^20^ and, in an early animal model, altered lung^21^ and brain cortex development with reversible, metabolite accumulation in brain lipidome of mouse pups.^18 22^ Additionally, there have been case reports of raised intracranial pressure in children with CF who have had direct administration of CFTRm.^23^ Systematic data, particularly regarding breastfeeding exposure, are also lacking. Despite these uncertainties, approximately 90% of FwCF elect to continue CFTRm therapy during pregnancy, as cessation has been associated with significant maternal health decline.^24^,^26^

At present, there is variation in the indication, scope and/or timing of clinical monitoring for infants born to FwCF who are exposed to CFTRm in utero and/or through breastfeeding, if they do not have CF themselves (infants who are diagnosed with CF are expected to receive routine specialist follow-up). Most CFTRm-exposed offspring will be non-CF obligate carriers of a maternal *CFTR* variant in whom the long-term implications of early CFTRm exposure (either transplacental or indirectly via breastfeeding) remain unclear. Current recommendations, based on limited observational data, focus primarily on ophthalmological screening for cataracts^27^ and serial liver function testing in infants with ongoing exposure via breastmilk;^28^ however, these practices are neither standardised nor consistently implemented in routine care.^29^ A recent Delphi consensus document does suggest recommendations for this follow-up, but it is too early to expect these to have been widely adopted into clinical practice.^30^

An additional concern arises from rare but clinically significant reports of false-negative CF newborn screening due to low immunoreactive trypsinogen (IRT) levels.^31^ IRT is produced by the pancreas, and in CF, pancreatic duct obstruction leads to its leakage into the bloodstream, which enables its use as a first-line screening test. In utero exposure to CFTRm may partially restore CFTR function and improve pancreatic function, potentially lowering circulating IRT and producing a negative screen. This may delay CF diagnosis, impacting on initiation of clinical management and family counselling.

Robust prospective, longitudinal data capturing maternal and child outcomes are urgently needed to inform clinical decision-making and guideline development. This study addresses this gap by providing the first longitudinal assessment of outcomes in children born to FwCF in the CFTRm era, offering timely and critical evidence to guide pregnancy, postnatal monitoring and long-term follow-up practices.

### The Royal Brompton Hospital CF-Reproductive and Maternal Health Service

**Figure 2:**
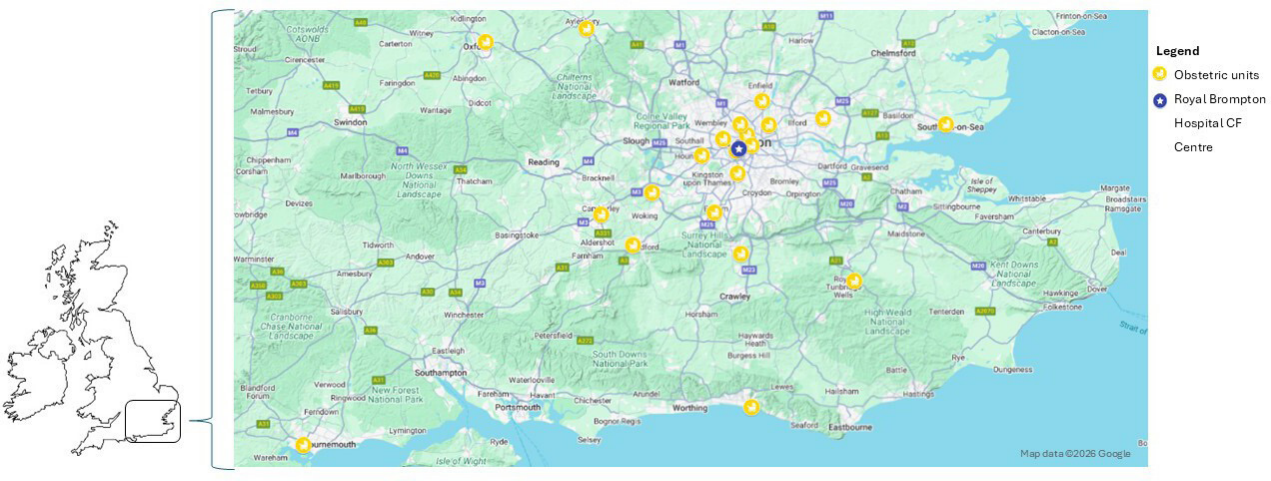
Map of obstetric unit geographic locations where FwCF cared for by the RBH-CF Reproductive and Maternal Health Service have given birth since 2021 (total: n=79).

The Royal Brompton Hospital (RBH), London hosts one of the largest adult CF centres in Europe, caring for over 600 adults with CF. In 2021, a unique CF-Reproductive and Maternal Health Service^32^ was established in response to its fourfold recorded increase in pregnancies following widespread ETI availability between 2019 and 2021.^25^ The service provides a dedicated specialist CF-Obstetric monthly multidisciplinary joint clinic, consisting of obstetricians, midwives and obstetric physicians from the linked maternity centre (Chelsea and Westminster Hospital) with CF-specialist physicians, nurses, physiotherapists, dietitians and pharmacists. Preconception counselling is offered for PwCF and their partners, with ongoing oversight and support of CF-maternal care during the antenatal period, and has recently introduced specialised postnatal maternal–infant dyad clinic reviews with paediatricians. The virtual outreach clinic model facilitates joint consultations between CF specialists, PwCF and more local obstetric teams when clinically appropriate, enabling safe and supported care in obstetric units geographically distant from the CF centre but close to home and family support for FwCF. Figure 2 shows the location of the CF centre in relation to obstetric units in the south of the UK where FwCF cared for by the RBH-CF Reproductive and Maternal Health service have given birth since 2021.

## Patient and public involvement

From its inception, the RBH-CF Reproductive and Maternal Health Service has formed strong patient–partner relationships with service users and has evolved and adapted rapidly in response to their feedback. A series of service evaluation interviews formed the basis of the research concept for this study and determined key research questions,^33 34^ which were further refined in dedicated focus groups throughout the protocol development process. Statements from the focus group included comments on wanting wider understanding of CF pregnancy among the non-CF HCPs and particularly better understanding of the personal risks of breastfeeding and impact on their infant.

A person with CF who is also a midwife is a named coinvestigator and facilitated subsequent patient information sheets, consent forms and protocol reviews. Additionally, a focus group of FwCF cared for at the RBH was convened to review study design of all three substudies to provide input into feasibility and acceptability of the study investigations and schedule. This group included FwCF who were planning to conceive, pregnant at the time or had previously been pregnant to capture a range of views and experiences.

The study forms part of a wider research consortium supported through the UK CF Trust strategic research centre grant (MATRIARCH SRC)^35^ which, in addition to scientific peer review, enabled patient and public involvement (PPI) panel feedback at grant application submission. Ongoing engagement with the CF Trust PPI groups, which include members of the public, PwCF and family members and/or carers of PwCF, has enabled further reach and involvement, including podcasts^36^ and national broadcast channels.^37^

## Methods and analysis

**Figure 3:**
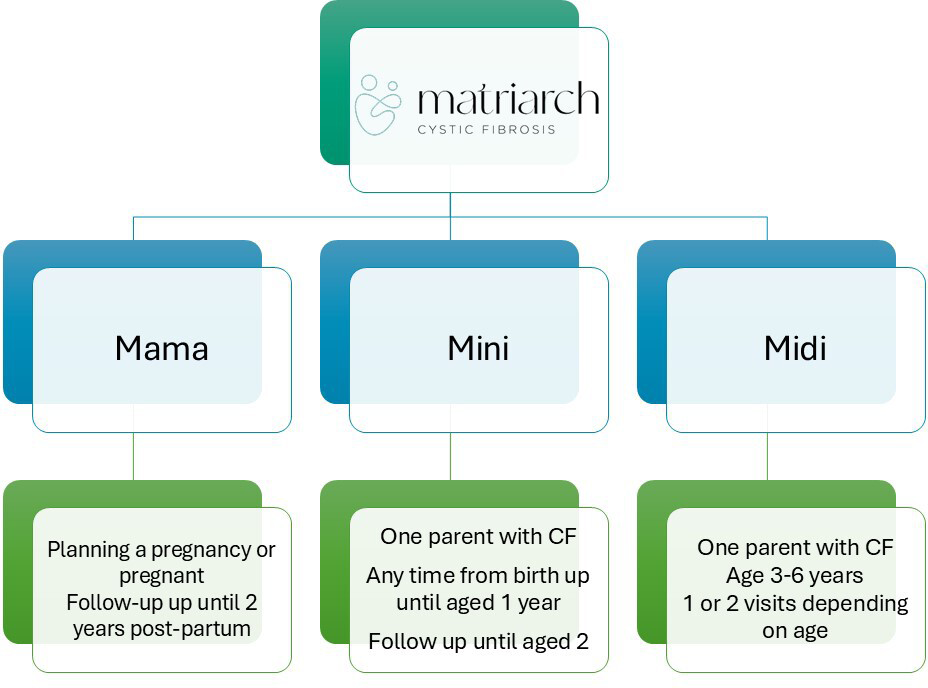
Study visit schedule for 'Mama’, 'Mini’ and 'Midi’. CF, cystic fibrosis

MATRIARCH_CF is a prospective, observational, mixed-methods study, comprising three linked substudies entitled: ‘Mama’, ‘Mini’ and ‘Midi’. A single, over-arching study protocol is warranted due to a continuation of participants, themes and data-sharing involving pregnant subjects (‘Mama’) and subsequent in utero environment for their offspring (‘Mini’ and ‘Midi‘) (figure 3).

Online supplemental appendix 1 presents the schedule of assessments for each substudy, detailing the procedures performed at each study visit.

### Substudy ‘Mama’ (preconception, pregnancy and postpartum)

Participants are recruited from the RBH-CF Reproductive and Maternal Health Service, either once they have stated an intention to conceive within the next 12 months or during their pregnancy, with follow-up until 2 years postpartum.

**Figure 4:**
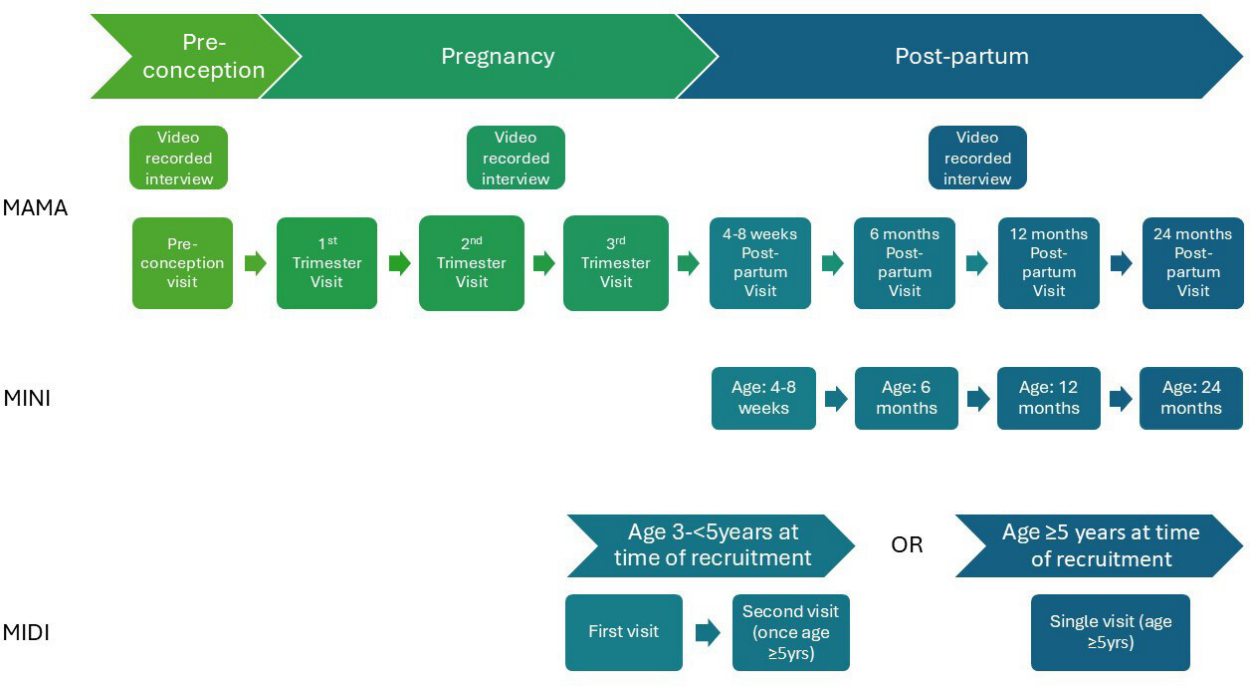
Study visit schedule for 'Mama', 'Mini' and 'Midi'.

There are up to eight in-person study visits and consent for data from additional clinical contacts is sought (figure 4). In-person study visits occur:

- Once preconception.
- Once per trimester of pregnancy.
- At four defined intervals post-partum.

Visits involve blood tests, lower respiratory tract samples, urine samples, spirometry, health and quality of life questionnaires, biological samples for CFTRm assay (blood and breastmilk) and sweat chloride measurements.

Continuous outcome data (CF-specific, obstetric and neonatal) are collected from both virtual and in-person clinical visits. This includes results of oral glucose tolerance tests if performed as standard of care.^38 39^ Consent will include permission to access and record clinical data collected prior to enrolment.

Radiological imaging occurs at three timepoints: a synchronous thoracic CT and oxygen-enhanced (OE)-MRI during preconception; thoracic OE-MRI and fetal MRI in the third trimester; repeat synchronous thoracic CT and OE-MRI at 6–12 months postpartum (no CT if participant is breastfeeding).

Semistructured video interviews with participants are offered and recorded for thematic analysis:

- Once during preconception.
- Once antepartum.
- Once 6–12 months postpartum

### Substudy ‘Mini’ (offspring 0–24 months)

Participants will have one biological parent with a confirmed diagnosis of CF. Children with in utero and/or breastfeeding exposure to CFTRm (ie, maternal CFTRm use during pregnancy and/or breastfeeding) will be recruited alongside non-exposed children to compare outcomes between groups. Given the high uptake of CFTRm during pregnancy among PwCF, we also seek to recruit children born to fathers with CF into the non-exposed comparator group. Sequential enrolment in ‘Mama’ and ‘Mini’ is possible, although recruitment occurs independently to each sub-study.

For children exposed to CFTRm in utero, visits take place concurrently with maternal clinical visits as per local Standard Operating Procedures (SOP) for the RBH maternal–infant dyad clinic. As part of this SOP, *CFTR* DNA testing is considered depending on the biological father’s *CFTR* genetic results. This is not mandated by the research protocol, but results of *CFTR* DNA testing performed as part of clinical care are collected for the study data set. All infants in the UK undergo routine national newborn screening, including IRT measurement, and the raw IRT value is collected for the study data set.

For unexposed infants, all visits and investigations are solely for research purposes. The study schedule also aligns with postpartum visits in ‘Mama’ to reduce study burden on dual-recruited families.

There are up to four in-person study visits within the first 2 years of life (figure 4). Visits are conducted by a paediatrician and include:

- Medical history and review of birth details (including *CFTR* genetic testing and ophthalmology screening result where clinically performed).
- Physical examination.
- Neurodevelopmental milestone assessments.
- Clinical laboratory tests including
  - Liver function tests (aspartate aminotransferase, alanine transaminase, gamma-glutamyltransferase, alkaline phosphatase and bilirubin).
  - Sweat chloride.
  - Faecal elastase.

Further investigations not currently part of the RBH-CF Maternal–Infant Dyad clinical schedule include the following:

- Cranial ultrasound (performed by specialist radiologist).
- Dried blood spot for CFTRm-assay validation.

### Substudy 'Midi' (offspring 3–6-year-olds)

Participants are children aged 3–6 years who have one biological parent with a diagnosis of CF. Recruitment and exposure grouping (CFTRm exposed and non-exposed) will follow the approach described in the ‘Mini’ substudy. All investigations will be offered irrespective of in utero and/or breastfeeding exposure to CFTRm. Children enrolled in ‘Mini’ will also be eligible to enrol in ‘Midi’ once they reach the appropriate age.

The study visit is conducted by a paediatrician and includes the following:

- Medical history and review of birth details.
- Physical examination.
- Neurodevelopmental milestone assessments.
- Multiple breath wash-out test to calculate Lung Clearance Index (LCI) with short extension.^40^
- Sweat chloride concentration.
- OE-MRI (NB: to avoid the requirement for sedation in young children, this is only offered to participants aged over 5 years; participants enrolled under the age of five are offered a second visit once over 5 years of age (figure 4)).

## Recruitment and sample size

Recruitment is pragmatic and designed to maximise participant numbers without a pre-determined sample size.

Although the increase in pregnancy rates in the UK population has been significant, overall numbers remain small and below the values that would be required to have an adequately powered sample to detect an overall significant difference in the primary outcome measure in the ‘Mama’ substudy of FEV_1_ change from preconception to postpartum, even if national recruitment were possible.

This study is focused on building a data set with high-resolution detail regarding participants and outcomes, including a rich and novel qualitative aspect, with results that complement larger population studies and better inform individual decision making.

Eligibility criteria are displayed in table 1.

**Table 1:** Eligibility criteria of each substudy

	Eligibility criteria
Mama	Under the care of Royal Brompton Hospital CF Reproductive and Maternal Health Service. Age 16 years or above at time of recruitment. Confirmed diagnosis of CF. Planning a pregnancy within the next 12 months or pregnant.
Mini	Infants who have a biological parent with a confirmed diagnosis of CF under the care of Royal Brompton Hospital Adult CF Service. Less than 12 months of age at first visit.
Midi	Children who have a biological parent with a confirmed diagnosis of CF who is under the care of Royal Brompton Hospital Adult CF Service. Age 3–6 years at time of first visit.

### ‘Mama’

Participants are recruited either in the preconception phase, defined as those intending or contemplating pregnancy within the next 12 months, or at any point during pregnancy to maximise this recruitment rate and include data from those undergoing unplanned pregnancies, an important cohort.

Using existing RBH data on pregnancy rates and focus group discussions, anticipated recruitment is eight participants per year of study.

### ‘Mini’

Infants are recruited from birth until 12 months of age. Participants can have a female or male biological parent with CF, although recruitment is expected to be less frequent in offspring of male parents with CF.

Anticipated recruitment is similar to ‘Mama’ with eight participants per study year.

### ‘Midi’

Children are recruited between the ages of 3 to 6 years old. Total recruitment is anticipated to be 20 participants.

## Endpoints and outcomes

Primary outcomes are summarised in table 2.

**Table 2:** Primary endpoints of each substudy

	Primary outcome measures
‘Mama’	Change in per cent predicted forced expiratory volume in 1 second (ppFEV_1_) from preconception/baseline to end of pregnancy, and at 12 and 24 months postpartum. Incidence of CF-related pulmonary complications during pregnancy. Incidence of premature delivery (defined as birth<37 weeks gestation).
‘Mini’ and ‘Midi’	The number of participants with: Liver dysfunction (defined as ALT/AST/GGT/bilirubin above upper limit of normal). This is prospectively obtained in the ‘Mini’ substudy and retrospectively reviewed from medical records in the ‘Midi’ substudy. Presence of congenital abnormalities including cataracts (based on medical history and examination). The number of participants with normal IRT subsequently diagnosed with CF. The number of participants with an abnormal Lung Clearance Index (‘Midi’ only)

ALT, alanine transaminase; AST, aspartate aminotransferase; CF, cystic fibrosis; GGT, gamma-glutamyltransferase; IRT, immunoreactive trypsinogen.

### Exploratory outcomes—Mama

#### Impact of pregnancy on pulmonary health

1. Incidence of pulmonary exacerbations during pregnancy and within first 24 months postpartum requiring oral and/or IV antibiotics.
2. Ventilation heterogeneity during and postpregnancy as measured by OE-MRI.
3. Change in haemoptysis frequency from baseline, and during pregnancy (as per number of episodes per person-years, including bronchial artery size assessment (by MRI) at baseline/preconception, antenatal and postpartum (6–12 months).

While ppFEV_1_ remains a key marker for overall lung health and severity of disease in PwCF, there are other important indicators both of overall health, but also of relevance when providing individual tailored preconception counselling for pregnancy and parenthood.

Functional impacts of pregnancy on pulmonary health will be explored by using both ppFEV_1_ and OE-MRI. The novel application of OE-MRI as an emerging technique to assess functional disease in PwCF^41^ may also be of increased sensitivity in the CFTRm era in comparison to traditional spirometry.^42^

#### Impact of pregnancy and parenthood on nutritional health

1. Change in weight from preconception until birth.
2. Change in weight according to breastfeeding status in first 24 months postpartum.
3. Change in fat-soluble vitamin levels during pregnancy and postpartum.

Weight and/or Body Mass Index (BMI) is an important survival marker in PwCF, and consideration of the impact of both caring responsibilities for a young family and the choice of infant feeding method will benefit from dedicated evaluation.

Weight gain is a requisite goal in healthy pregnancies. The Institute of Medicine recommends a total weight gain of approximately 10 kg for females with a normal BMI at the start of pregnancy^43^ and greater for those who are underweight. Pregnancies in females who are underweight are associated with a greater risk of small for gestational age infants or preterm birth.^43^ Given the association of poor nutritional status for PwCF, particularly those who have pancreatic exocrine insufficiency (80% of adults with CF in the UK require pancreatic enzyme replacement),^5^ pregnancy weight-gain outcomes will provide further insight into factors contributing to increased rates of these complications in FwCF.

Due to exocrine pancreatic insufficiency, PwCF often need fat-soluble vitamin supplementation. Vitamin A levels, in particular, are of utmost importance during pregnancy as excess levels lead to teratogenic effects. Monitoring of fat-soluble vitamins will provide further insight into supplementation practices during pregnancy and breastfeeding.

#### Impact of pregnancy on CFTRm efficacy and CFTR function

1. Sweat chloride levels during pregnancy and variability per trimester.

Sweat chloride is a validated clinical and research surrogate marker of CFTR function.^44^ Pregnancy affects metabolism, circulating blood volume and medication pharmacokinetics. The observation of sweat chloride variability through pregnancy will reflect how CFTR function may also be affected through gestational changes in FwCF and may be of particular relevance for those taking CFTRm. This may subsequently guide individualised CFTRm dosing during pregnancy, a strategy already considered in some European centres through therapeutic drug monitoring.^45^

### Obstetric outcomes of FwCF

1. Miscarriage and terminations.
2. Caesarean section rate.
3. Incidence of birth expedited by induction or Caesarean section for maternal pulmonary indications.
4. Incidence of spontaneous preterm birth.
5. Incidence of pregnancy-specific medical conditions including intrahepatic cholestasis, pregnancy-induced hypertension and pre-eclampsia.
6. Incidence of gestational diabetes.
7. Incidence of large- or small-for-gestational-age infants.
8. Obstetric outcomes according to diabetes status and therapy.
9. Breastfeeding duration and health impact

During pregnancy, FwCF in the UK are under the care of both their obstetric team and their CF centre. Understanding the specific obstetric risks and outcomes will provide important information to risk stratify pregnancies in terms of optimal care-setting and category of maternal medicine network^46^ support and also aid obstetricians less familiar with CF as a whole in decision making around place and timing of delivery and management of complications.

#### Psychological impact of pregnancy and parenthood

1. Change in quality of life and mental health/perinatal mental health scores (CFQ-R,^47^ EQ-5D-5L,^48^ PHQ9^49^ and GAD7).^50^
2. Incidence of postnatal anxiety/depression.
3. Thematic analysis of experiences of females with CF from data collected through semistructured interviews throughout:
  - Trying to conceive.
  - Pregnancy and childbirth.
  - Early parenthood.

The impact of considering and experiencing pregnancy in the context of managing a long-term, hereditary, life-limiting condition means FwCF enter pregnancy and parenthood with a unique lens of life experience and future considerations.

There are studies showing that perception of risk and experience of pregnancy can be different in people with a chronic disease,^51 52^ but all CF-specific studies^53^ are retrospective. Exploring and recording the thoughts, experiences and considerations of FwCF contemporaneously will avoid the impact of outcome bias, particularly preconception, allow for capture of both positive and negative experiences including fetal loss/miscarriage/terminations and be of value guiding future understanding of each key stage.

### Exploratory outcomes—Mini and Midi

#### Outcomes specifically related to CFTRm exposure

1. Frequency of raised blood pressure (>95th centile for age, sex and height on two separate readings).
2. Number of participants with raised intracranial pressure diagnosed on cranial ultrasound (‘Mini’ only).
3. Presence of any predefined structural abnormality on structural lung MRI and/or functional abnormality on OE-MRI (‘Midi’ only).

The above outcomes refer specifically to possible concerns raised either in animal models (eg, abnormal lung development reported in in vitro studies which involved CFTRm exposure in mouse models)^21^ or case series (hypertension,^54^ raised intracranial pressure),^23^ but the relevance in non-CF children has not yet been studied.

This will be the first dedicated study of these possible effects in CFTRm exposed offspring, and although not powered to safely exclude side-effects, positive findings would be of material importance for the CF community.

#### Child development outcomes

1. Number of participants with below neonatal birth outcomes:
  - Prematurity (defined as being born <37 weeks’ gestation).
  - Low birth weight <2nd centile.
  - Special care or neonatal intensive care unit admission.
2. Frequency of faltering growth (as per NICE guidelines [NG75]).^55^
3. Frequency of delayed development, defined as failure to attain milestones by the upper limit of the expected age range, across the gross motor, vision and fine motor, hearing, speech and language, and social domains.

Very few medications used for chronic health conditions in pregnancy are prospectively monitored among the offspring until beyond infancy and into childhood. Signal for harm is non-systematically gathered via ad hoc reporting systems such as the Medicines and Healthcare products Regulatory Agency (MHRA) yellow card systems^56^ or overall population exposure. Given the small numbers on a population level of children exposed to CFTRm, this method of detecting longer term side effects is unreliable at best and so given the rare opportunity to actively review children years following in utero exposure, a full clinical assessment is of benefit here.

## Statistical analysis

### Primary outcomes analysis

A descriptive analysis of the outcomes will be completed. For categorical variables (eg, presence of cataracts), they will be summarised as frequencies (%). For continuous variables (eg, weight centiles), they will be reported as a mean (SD) or median (IQRs) dependent on sample distribution.

For comparisons across groups, a chi-square or Fisher’s exact test will be used for categorical variables and a two-sample t-test or Mann-Whitney U test for continuous variables. Confounders including but not limited to cumulative time of exposure to CFTRm, maternal health and gestation of offspring will be considered.

In the ‘Mini’ substudy, enrolling offspring for their first visit up to the age of 12 months allows for maximising sample size although resulting in the possibility of missing early life events. A sensitivity analysis to test assumptions such as only including those up to 6 months of age will be undertaken.

### Exploratory outcome analysis

A descriptive analysis of the exploratory outcomes will be undertaken as per the approach adopted for the primary endpoint analysis described in the ‘Exploratory outcomes—Mama’ section.

The interviews in the ‘Mama’ sub-study will be pseudo-anonymised, recorded and transcribed live within MS Teams, with anonymised transcripts imported into Nvivo software.^57^ The interviews will be analysed in this programme using thematic analysis, in a structure similar to that described by Braun and Clarke.^58^ Interviews will be undertaken in an iterative manner where analysis occurs synchronously alongside data collection, allowing interview schema to be revised as new themes evolve which require further exploration as per established qualitative methodology.^59 60^ To increase the robustness, theme checking will be performed by members of the study team, and member checking will be performed by participants prior to publication.^59 60^

## Ethical considerations and dissemination

The exclusion of pregnant and lactating FwCF from phase 2 and 3 trials of CFTRm means that recent data relating to outcomes of FwCF pregnancies and their offspring is limited to retrospective case series in the modern therapeutic era. This exclusion is common across most clinical trials. The WHO have highlighted the importance of appropriate inclusion of pregnant and breastfeeding individuals in clinical research.^34^ The WHO have described an imbalance in pregnancy-related research in which potential harms are prominently considered (denoted as ‘risk sensitivity’), while the benefits of generating evidence are undervalued (‘benefit insensitivity’). From an ethical perspective, it is therefore important to generate high-quality data to strengthen the evidence base, resolve bias and support informed clinical decision-making during pregnancy and breastfeeding.

The decision to continue CFTRm without licence during pregnancy for the majority of FwCF^15 26 61^ is a decision with significant impacts for both the expectant mother and their healthcare team regarding the ethical and emotional burden of shared decision making, in the absence of a more robust, salient evidence base and longer term safety data. Ceasing or pausing such a critical treatment for FwCF for 9 months or longer requires careful consideration, particularly given that case series evidence to date shows no signal for major harm in infants exposed in utero or via breastmilk but with established association with significant maternal health destabilisation.^19 62^ Dedicated longer term monitoring of these offspring through this study will provide additional information to inform future guidelines for follow-up and investigation of these infants, and crucially also be of relevance in the individualised decisions made by FwCF relating to how their own CF-health may intersect and impact that of their children.

As with many medications used during pregnancy and lactation, CFTRm are unlicensed in this context. As an observational study, all discussions and decision making around continuing or ceasing CFTRm use will be performed by the clinical team and not be influenced by study enrolment.

Results of this study will be submitted as part of doctoral theses and for publication in peer reviewed journals. They will also be submitted to relevant national and international conferences.

This study has been reviewed by the NHS ethics HSC REC A committee and given ethical and HRA approval (25/NI/0027 19 March 2025).

## Supplementary material

10.1136/bmjresp-2026-004270 online supplemental file 1

10.1136/bmjresp-2026-004270 online supplemental file 2

## Data Availability

No data are available.
