# Supplementary material for: Maternal, Infant, Reproductive and Child Health in Cystic Fibrosis (MATRIARCH_CF): a prospective, observational study to evaluate pregnancy and parenthood in females with cystic fibrosis and health of the offspring in the CFTR-modulator era
Source: BMJ Open Respir Res. 2026 Jun 30;13(1):e004270. doi: 10.1136/bmjresp-2026-004270 (PMC13331013; doi:10.1136/bmjresp-2026-004270)
Supplement: online supplemental file 1 [file bmjresp-13-1-s001.docx]

# Appendix 1

| Study Procedure | Pre-conception | 1^st^ trimester | 2^nd^ trimester | 3^rd^ trimester | 4-8 weeks PP | 6 months PP | 12 months PP | 24 months PP |
| --- | --- | --- | --- | --- | --- | --- | --- | --- |
| Informed consent | X |  |  |  |  |  |  |  |
| Inclusion/exclusion criteria | X |  |  |  |  |  |  |  |
| Demographics | X |  |  |  |  |  |  |  |
| Sputum sample | X | X | X | X | X | X | X | X |
| Clinical Blood tests^2^ | X | X | X | X | X |  | X | X |
| Blood test for CFTRm assay | X | X | X | X | X | X | X | X |
| PAPP-A and HCG |  | X |  |  |  |  |  |  |
| Urine sample | X | X | X | X | X |  |  |  |
| Lung function | X | X | X | X | X | X | X | X |
| Sweat test | X | X | X | X | X |  |  |  |
| Lung CT^1^ | X |  |  |  |  | X | |  |
| Lung MRI^1^ | X |  |  | X |  | X | |  |
| Fetal MRI |  |  |  | X |  |  |  |  |
| EPDS^3^ |  |  |  | X | X |  |  |  |
| Questionnaires^4^ | X | X | X | X | X | X | X | X |
| Semi-structured interview | X |  | X | |  | X | |  |
| Breast milk sample for CFTRm assay^5^ |  |  |  |  | X | X | X |  |
| Key:  X: Planned completion date   1. Lung Imaging – CT will only be performed on non-lactating and non-pregnant participants. Post-partum CT and imaging will be paired, so either a CT and MRI done at the same visit if no longer breastfeeding, or a single MRI if still breastfeeding at 1 year 2. Clinical blood tests – Full blood count, renal profile, liver function, Vitamin A, D, E and K, CRP, OGTT if clinically relevant, HbA1c 3. EPDS – Edinburgh Postnatal Depression Score 4. Questionnaires to include CFQ-R, EQ-5D-5L, PHQ9 and GAD7 5. If breastfeeding whilst taking CFTRm | | | | | | | | |

### Mama Sub-study

### Mini Sub-study

| Study Procedure | Visit 1  4-8 weeks age | Visit 2  6 months of age | Visit 3  12 months of age | Visit 4  24 months of age |
| --- | --- | --- | --- | --- |
| **For all children enrolled** | | | | |
| Informed consent | X |  |  |  |
| Inclusion/exclusion criteria | X |  |  |  |
| Demographics | X |  |  |  |
| Baseline assessments^1^ | X |  |  |  |
| Medical History^2^ | X | X | X | X |
| Physical examination^3^ | X | X | X | X |
| Liver function blood tests^4^ | X | X | X^5^ | X^5^ |
| Sweat test^6^ | X | X^7^ |  |  |
| Faecal elastase^8^ | X | X^7^ |  |  |
| Cranial ultrasound^9^ | X |  |  |  |
| **Only for children exposed to CFTRm** | | | | |
| Document ophthalmological review^10^ | X |  | X^5^ | X^5^ |
| CFTRm assay^11^ | X | X | X^5^ | X^5^ |
| **Key**:  X: Planned completion date  Highlighted in grey: tests that are additional to standards of practice. Note that all tests for children not exposed to CFTRm are additional to standards of practice.  1. See section 9.4.2  2. See section 9.4.4.  3. See section 9.4.5.  4. See section 9.4.6  5. Only if breastfed and exposed to CFTR modulator in last 6 months  6. See section 9.4.8  7. If exposed to CFTRm via breast feeding at the 4 – 8 weeks visit to repeat at 6 months. If still exposed at 6 months repeat at 12 months and so on, as exposure to CFTRm via lactation could affect results  8. See section 9.4.9.  9. See section 9.4.10  10. See section 9.4.3  11. See section 9.4.7 | | | | |

### Midi sub-study

| Study Procedure | Age 3 - <5 years at time of recruitment | | Age 5+ at time of recruitment |
| --- | --- | --- | --- |
|  | First visit | Second visit (once aged 5+) | Single visit |
| Informed consent | X |  | X |
| Inclusion/exclusion criteria | X |  | X |
| Demographics | X |  | X |
| Baseline assessments^1^ | X |  | X |
| Medical history^2^ | X | X | X |
| Physical examination^3^ | X | X | X |
| Sweat test^4^ | X |  | X |
| O2 enhanced Lung MRI^5^ |  | X | X |
| Lung clearance index^6^ | X | X | X |
| **Key**:  X: Planned completion date  1. See section 9.4.2  2. See section 9.4.4.  3. See section 9.4.5.  4. See section 9.4.8  5. See section 9.4.12  6. See section 9.4.11 | | | |
