# Supplementary material for: Maternal, Infant, Reproductive and Child Health in Cystic Fibrosis (MATRIARCH_CF): a prospective, observational study to evaluate pregnancy and parenthood in females with cystic fibrosis and health of the offspring in the CFTR-modulator era
Source: BMJ Open Respir Res. 2026 Jun 30;13(1):e004270. doi: 10.1136/bmjresp-2026-004270 (PMC13331013; doi:10.1136/bmjresp-2026-004270)
Supplement: online supplemental file 2 [file bmjresp-13-1-s002.docx]

STUDY PROTOCOL


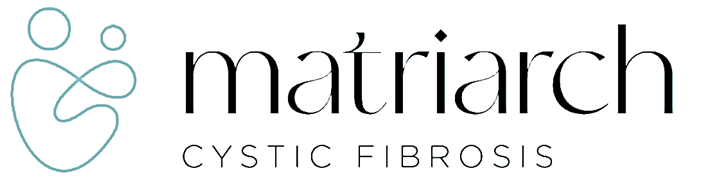


**An observational study to evaluate the impact of pregnancy and parenthood in females with Cystic Fibrosis and their offspring in the CFTR modulator era.**

**Short Study:** Maternal, Infant, Reproductive & Child Health in Cystic Fibrosis **- MATRIARCH_CF**

**IRAS Project ID:** 352398

**Sponsor’s Details:** Royal Brompton Hospital, Sydney Street, London, SW3 6NP

**CHIEF INVESTIGATOR (CI):**

Dr Imogen Felton

Consultant in Respiratory Medicine & Adult Cystic Fibrosis,

Address: Royal Brompton Hospital

Phone: 020 7352 8121 x84233

Email [i.felton@rbht.nhs.uk](mailto:i.felton@rbht.nhs.uk)

**LEAD CO-ORDINATING INVESTIGATOR (LCI)**

Professor Jane Davies

Professor of Paediatric Respirology & Experimental Medicine NHLI

Honorary Consultant in Paediatric Respiratory Medicine, Royal Brompton Hospital, Guy’s & St Thomas’ Trust

Address: Imperial College London, NIHR Biomedical Research Centre

Phone: 020 7352 8121 x88398

Email: [j.c.davies@imperial.ac.uk](mailto:j.c.davies@imperial.ac.uk)

**SPONSOR REPRESENTATIVE:**

Ira Jakupovic, Head of Research Governance and Regulatory Compliance Royal Brompton and Harefield Hospitals

Royal Brompton Hospital (RBH)

Research Office, Sydney Street

London SW3 6NP

Email: [i.jakupovic@rbht.nhs.uk](mailto:i.jakupovic@rbht.nhs.uk)

Information in this protocol is confidential and should not be disclosed, other than to those directly involved in the execution or the ethical/regulatory review of the study, without written authorisation from RBHH Research Office (RO) or its affiliates.

# Signature Page and Statement

The Chief Investigator (CI) and the sponsor representative have discussed this protocol. The investigators agree to perform the investigations and to abide by this protocol except in the case of medical emergency or where departures from it may be mutually agreed in writing.

The Investigator agrees to conduct the study in compliance with the protocol, GCP, the Data Protection Act (1998), the Trust Information Governance Policy, the UK Policy Framework for Health and Social Care Research (as amended), the Sponsor’s SOPs, and other regulatory requirements as appropriate.

This protocol has been written in accordance with the Sponsor’s procedure for writing study protocols outlining study procedures for the conduct and management of studies sponsored by RBHH, part of GSTT.

| **Chief Investigator (CI)** | 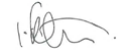 | 20/01/2025 |
| --- | --- | --- |
| Imogen Felton, Consultant in Respiratory Medicine & Adult Cystic Fibrosis |  |  |
| Royal Brompton and Harefield Hospitals (RBHH) | Signature | Date |
| **Lead co-ordinating investigator (LCI)** |  |  |
| Jane Davies, Professor of Paediatric Respirology & Experimental Medicine at NHLI | 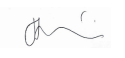 | 20/01/2025 |
| Imperial College London | Signature | Date |
| **Sponsor Representative** |  |  |
| Ira Jakupovic, Head of Research Governance and Regulatory Compliance |  |  |
| Royal Brompton and Harefield Hospitals (RBHH) | Signature | Date |

# Contents

[Signature Page and Statement 2](#_Toc1412040674)

[Contents 3](#_Toc453411322)

[1 List of abbreviations 6](#_Toc1965799540)

[2 Study personnel 7](#_Toc1961170569)

[3 Study synopsis 8](#_Toc1239776104)

[4 Introduction 11](#_Toc1858909875)

[4.1 Background 11](#_Toc982939137)

[4.2 Pre-clinical and clinical data 12](#_Toc1623023223)

[4.2.1 Pre-clinical data 12](#_Toc1017375467)

[4.2.2 Clinical data 13](#_Toc680236354)

[4.3 Study Rationale and risk/benefit analysis 14](#_Toc158897441)

[4.3.1 Study Rationale: 14](#_Toc1426518225)

[4.3.2 Risks: 14](#_Toc2121395956)

[4.3.3 Benefits: 15](#_Toc1051244886)

[4.4 Management of potential study risks 15](#_Toc712583012)

[5 Study Aims and Objectives 16](#_Toc1876522712)

[5.1 Primary aims 16](#_Toc1244264432)

[5.2 Primary Objectives 16](#_Toc67827822)

[5.3 Exploratory Objectives 16](#_Toc1190852577)

[6 Study design 17](#_Toc1967700793)

[6.1 Overall design 17](#_Toc1886921315)

[6.2 Schematic of study design 20](#_Toc1680048768)

[7 Eligibility criteria 21](#_Toc1934061306)

[7.1 Inclusion criteria 21](#_Toc1975245400)

[7.2 Exclusion criteria 21](#_Toc757820158)

[8 Recruitment process 21](#_Toc521650755)

[9 Study procedures and assessments 22](#_Toc1605589996)

[9.1 Informed consent 22](#_Toc1962045654)

[9.2 Screening assessments 23](#_Toc532788208)

[9.3 ‘Mama’ sub-study assessments 23](#_Toc1315995193)

[9.3.1 Summary flow chart of study assessments (Table 1) 23](#_Toc1239830927)

[9.3.2 Baseline assessments 24](#_Toc485549986)

[9.3.3 Medical History 25](#_Toc979660465)

[9.3.4 Sweat test 25](#_Toc356777449)

[9.3.5 Blood tests 25](#_Toc1018077272)

[9.3.6 Breastmilk sample 25](#_Toc251006369)

[9.3.7 Qualitative Interviews 25](#_Toc603783594)

[9.3.8 Spirometry 25](#_Toc1137715622)

[9.3.9 Sputum samples 25](#_Toc499782789)

[9.3.10 Urine sample 26](#_Toc2009681539)

[9.3.11 CT scan 26](#_Toc14854550)

[9.3.12 Oxygen-enhanced Magnetic Resonance Imaging (OE-MRI) of lungs 26](#_Toc1440144578)

[9.3.13 Fetal MRI 26](#_Toc1278420015)

[9.4 ‘Mini’ and ‘Midi’ sub-study assessments 26](#_Toc1687458587)

[9.4.1 Summary flow chart of study assessments (Table 2 & 3) 26](#_Toc1942511424)

[9.4.2 Review of medical records (baseline assessment) 28](#_Toc1670390284)

[9.4.3 Documentation of ophthalmology review 29](#_Toc120383055)

[9.4.4 Medical history 29](#_Toc1114855548)

[9.4.5 Physical examination 29](#_Toc18778748)

[9.4.6 Liver function blood tests 29](#_Toc832976835)

[9.4.7 Blood test for CFTRm assay and serum save 30](#_Toc1040344503)

[9.4.8 Sweat chloride testing 30](#_Toc1677582045)

[9.4.9 Faecal elastase 30](#_Toc928639407)

[9.4.10 Cranial ultrasound (‘Mini’ study only) 30](#_Toc361982458)

[9.4.11 Lung clearance index (LCI) (‘Midi’ sub-study only) 30](#_Toc1741628928)

[9.4.12 Oxygen (O2)-enhanced Lung MRI (‘Midi’ sub-study only) 30](#_Toc235687007)

[10 Definition of the End of Study 31](#_Toc1230447629)

[11 Discontinuation/withdrawal of participants and stopping rules 31](#_Toc1051797400)

[12 Safety Reporting 31](#_Toc178355872)

[12.1 Definition 31](#_Toc31417649)

[12.2 Recording Adverse Events (AEs) 31](#_Toc101772240)

[12.3 Assessment of SAEs 33](#_Toc671114148)

[12.4 Expected AEs 33](#_Toc920116594)

[12.5 Reporting of SAEs to the sponsor and the REC 33](#_Toc638733364)

[12.6 The type and duration of Follow up 34](#_Toc1701178129)

[12.7 Annual Progress Reports (APRs) 34](#_Toc1165229608)

[12.8 Reporting Urgent Safety Measures 34](#_Toc1345266025)

[12.9 Notification of Serious Breaches of GCP and/or the protocol 34](#_Toc368322886)

[13 Data management and quality assurance 35](#_Toc640463843)

[13.1 Confidentiality 35](#_Toc1625620433)

[13.2 Data collection tool 35](#_Toc631618135)

[13.3 Data handling and analysis 35](#_Toc418267124)

[14 Archiving arrangements 36](#_Toc1262333943)

[15 Statistical design 36](#_Toc1195712148)

[15.1 Statistical input in study design 36](#_Toc1424447181)

[15.2 Endpoints 36](#_Toc865398363)

[15.2.1 Primary endpoints 36](#_Toc1642935150)

[15.2.2 Exploratory endpoints 37](#_Toc306356334)

[15.3 Sample size and recruitment 38](#_Toc527072434)

[15.3.1 Sample size calculation 38](#_Toc2010794210)

[15.3.2 Planned recruitment rate 38](#_Toc1820541138)

[15.4 Statistical analysis plan 39](#_Toc1259278789)

[15.4.1 Summary of baseline data and flow of patients 39](#_Toc598200862)

[15.4.2 Primary endpoint analysis 39](#_Toc664483315)

[15.4.3 Exploratory endpoint analysis 39](#_Toc199734098)

[15.5 Interim analysis 40](#_Toc2085421188)

[15.6 Other statistical considerations 40](#_Toc1088961960)

[16 Committees in involved in the study 40](#_Toc1032375169)

[16.1 Study Management Group 40](#_Toc103875151)

[16.2 Independent Advisory Group 40](#_Toc35905722)

[16.3 Patient and Public involvement 40](#_Toc1444686656)

[17 Direct access to source data 40](#_Toc1421455857)

[18 Ethics and regulatory requirements 40](#_Toc41185924)

[19 Monitoring plan for the study 41](#_Toc1532455207)

[20 Finance 41](#_Toc940391269)

[21 Insurance and indemnity 41](#_Toc621663942)

[22 Publication policy 41](#_Toc1173632526)

[23 Statement of compliance 41](#_Toc36947950)

[24 List of Protocol appendices 43](#_Toc1301985263)

[25 References 45](#_Toc1455542752)

# List of abbreviations

AE Adverse Event

AR Adverse Reaction

ASR Annual Safety Report

BMI Body Mass Index

CF Cystic Fibrosis

CFQ-R Cystic Fibrosis Questionnaire – Revised

CI Chief Investigator

CRF Case Report Form

DMC Data Monitoring Committee

EPDS Edinburgh Postnatal Depression Score

ETI Elexacaftor-Tezacaftor-Ivacaftor

EQ-5D-5L EQ-5D-5L (this is the full name of the questionnaire)

(pp)FEV_1_ (percentage predicted) Forced expiratory volume in 1 second

FVC Forced vital capacity

GAfREC Governance Arrangements for NHS Research Ethics

GCP Good Clinical Practice

HRA Health Research Authority

HRQOL Health related quality of life

ICF Informed Consent Form

IQR Interquartile range

ISF Investigator Site File

ISRCTN International Standard Randomised Controlled Trial Network

LCI Lung Clearance Index

LTOT Long term oxygen therapy

MCID Minimally clinically important difference

MIC Minimally important change

MBW Multiple Breath Washout

NICE National Institute for Health and Care Excellence

NHS R&D National Health Service Research & Development

PI Principal Investigator

PIS Participant Information Sheet

QA Quality Assurance

QC Quality Control

RBHH Royal Brompton and Harefield Hospitals

RCT Randomised Control Trial

REC Research Ethics Committee

SAE Serious Adverse Event

SAR Serious Adverse Reaction

SD Standard deviation

SDV Source Document Verification

SOP Standard Operating Procedure

SSA Site Specific Assessment

TMG Trial Management Group

# Study personnel

**Chief Investigator:**

Dr Imogen Felton

Consultant in Respiratory Medicine & Adult Cystic Fibrosis,

Address: Royal Brompton Hospital

Phone: 020 7352 8121 x84233

Email [i.felton@rbht.nhs.uk](mailto:i.felton@rbht.nhs.uk)

**Lead Co-ordinating Investigator:**

Professor Jane Davies

Professor of Paediatric Respirology & Experimental Medicine NHLI

Honorary Consultant in Paediatric Respiratory Medicine, Royal Brompton Hospital, Guy’s & St Thomas’ Trust

Address: Imperial College London, NIHR Biomedical Research Centre

Phone: 020 7352 8121 x88398

Email: [j.c.davies@imperial.ac.uk](mailto:j.c.davies@imperial.ac.uk)

**Routine local laboratories:**

Royal Brompton Hospital Clinical Biochemistry Laboratory
Email: [rbh-trbiochemistrynhsnet@rbht.nhs.uk](mailto:rbh-trbiochemistrynhsnet@rbht.nhs.uk)Phone: 020 7352 8121 ext 88411

# Study synopsis

| **Full study title:** | An observational study to evaluate the impact of pregnancy and parenthood in females with Cystic Fibrosis and their offspring in the CFTR modulator era. |
| --- | --- |
| **Short study title:** | MATeRnal, InfAnt, Reproductive & Child Health in Cystic Fibrosis (MATRIARCH_CF) |
| **IRAS ID number:** | **352398** |
| **Chief Investigator:** | Dr Imogen Felton |
| **Lead Co-ordinating Investigator** | Professor Jane Davies |
| **Study centres/sites:** | Royal Brompton Hospital (RBH), Guy’s and St Thomas’ Hospitals NHS Foundation Trust, Sydney St, London, SW3 6NP |
| **Study duration:** | Three years |
| **Primary Aims:** | 1. **Sub-study ‘Mama’**: To describe the impact of pregnancy and the first 12-24 months of parenthood in females with cystic fibrosis on their physical and psychological health. 2. **Sub-study ‘Mini’ & ‘Midi’:** To collect clinical data that will enable assessment of health outcomes in offspring of parents with CF in the short (up to 24 months) and medium-term (aged three-to-six years) |
| **Primary Objectives:** | 1. **Sub-study ‘Mama’**    1. To record the obstetric and neonatal outcomes of females with CF.    2. To record CF-related health outcomes among females with CF including pulmonary (e.g. ppFEV_1_) and nutritional status (e.g. BMI) during pregnancy and early parenthood (up to 24 months).    3. To record the subjective experiences of family planning, pregnancy and early parenthood in females with CF.    4. To describe the impact of diabetes (pre-existing and gestational) on obstetric, neonatal and CF related outcomes.    5. To record sweat chloride results during pregnancy and/or lactation among females with CF in relation to CFTR modulator therapy. 2. **Sub-studies ‘Mini’ and ‘Midi’**    1. To collate clinically obtained serum liver enzymes levels and to record the frequency of congenital abnormalities (including ophthalmic cataract screening), in children born to a parent with CF (females or males with CF).    2. To document cases of ‘missed’ CF-diagnoses due to false negative immunoreactive trypsinogen (IRT) results in children of females with CF in relation to transplacental and/or lactational exposure to CFTR modulators.    3. To record lung function indices using lung clearance index (LCI) of young children (aged three-to-six years) born to a parent with CF. |
| **Exploratory Objectives:** | 1. **Sub-study ‘Mama’**    1. To provide biological samples to facilitate validation of a measurable assay for the individual components of CFTR modulators in maternal blood and breastmilk.    2. To record maternal serum and urine hormonal and micro-nutrient changes through pregnancy.    3. To review lower respiratory tract microbiology in relation to pulmonary status (e.g. pulmonary exacerbation frequency and haemoptysis) among females with CF during pregnancy and early parenthood (up to 24 months).    4. To identify changes in maternal bronchial artery size during pregnancy with antenatal radiological imaging (MRI) and explore rates of haemoptysis in pregnant and post-partum females with CF.    5. To identify structural lung changes and functional impacts using oxygen-enhanced lung MRI (OEMRI) during pregnancy.    6. To document fetal radiological findings in relation to embryological ophthalmic and pulmonary development. 2. **Sub-studies ‘Mini’ and ‘Midi’**    1. To record blood pressure and cranial ultrasound measurements in children born to a parent with CF.    2. To record routine neonatal health outcomes, offspring growth and developmental milestones in children born to a parent with CF.    3. To record lung MRI results in young children (aged three-to-six years) born to a parent with CF.    4. To provide biological samples to facilitate validation of a measurable assay for the individual components of CFTRm in the blood of babies exposed to CFTRm in utero and/or via lactation. |
| **Study population:** | 1. Females aged 16 years or older with CF under the care of the Royal Brompton Hospital adult CF Unit who are planning a pregnancy or pregnant. 2. Biological offspring of people with CF (both mothers and fathers) cared for by the Royal Brompton Hospital Adult CF Service, from birth to the age of six years. |
| **Methodology:** | This is a non-commercial, observational mixed-methods study comprising three linked sub-studies named: **MATRIARCH_CF:** **‘Mama’, ‘Mini’, and ‘Midi’.**  A single, over-arching study protocol is warranted due to a continuation of participants, themes and data-sharing from pregnant subjects (‘Mama’) and subsequent in utero environment in their offspring (‘Mini’ and ‘Midi’).  As an observational study, all the assessments described below will be optional, and participants will not be excluded from the study if they wish to decline specific investigations.  ***Sub-study 1 – ‘Mama’ (pre-conception, pregnancy and parenthood)***  ‘Mama’ is a sub-study of pre-conception, pregnancy and parenthood in adult females with CF. This is a prospective, mixed-methods, observational cohort study, collecting data generated in a clinical setting through the local Royal Brompton Hospital CF Reproductive and Maternal Health Service.  ***Sub-study 2 –’Mini’ (Offspring 0 - 24 months)***  The ‘Mini’ sub-study is a prospective, observational cohort study of children who have at least one biological parent with a diagnosis of CF. It is expected that many, but not all, of these children will be those born to females participating in the ‘Mama’ sub-study.  There will be a maximum of four visits in two years, with visits including physical examination, specimen collection (blood, sweat, faeces), and imaging (cranial ultrasound).  ***Sub-study 3 –’Midi’ (Offspring 3 – 6-year-olds)***  ‘Midi’ is a cross-sectional, cohort sub-study of children aged three-to-six years who have at least one biological parent with a diagnosis of CF. Participants from ‘Mini’ will be eligible to progress into the ‘Midi’ protocol once the appropriate age has been reached.  There will be a maximum of two visits, with visits including physical examination, specimen collection (sweat), lung function testing and imaging (lung MRI). |
| **Eligibility criteria:** | ***Inclusion criteria – ‘Mama’ sub-study:***   - Written informed consent obtained from participant. - Under the care of Royal Brompton Hospital CF Reproductive and Maternal Health Service - Age 16 years or above at time of recruitment - Confirmed diagnosis of CF - Planning a pregnancy or pregnant at time of recruitment. - Ability to adhere to the required visits and investigations.   ***Inclusion criteria –’Mini’ sub-study:***   - Written informed consent obtained from participant’s legal guardian. - Infants who have a biological parent with a confirmed diagnosis of CF under the care of Royal Brompton Hospital Adult CF Service - Less than 12 months of age at first visit. - Ability to adhere to the required visits and investigations.   ***Inclusion criteria –’Midi’ sub-study:***   - Written informed consent obtained from participant’s legal guardian. - Children who have a biological parent with a confirmed diagnosis of CF who is under the care of Royal Brompton Hospital Adult CF Service. - Age three to six years at time of visit - Ability to adhere to the required visits and investigations. |
|  | ***Exclusion criteria – ‘Mama’ sub-study:***   - Any significant health condition which would cause inability to comply with protocol based on investigator discretion. - History of lung transplantation   ***Exclusion criteria – ‘Mini’ sub-study:***   - Legal guardians’ inability to provide consent to participate in the study. - Mother of infant has significant comorbidities unrelated to CF which could affect infant outcomes based on investigator discretion. - A significant comorbidity in the infant unrelated to CFTR modulator exposure which could affect their outcomes, based on investigator discretion.   ***Exclusion criteria – ‘Midi’ sub-study:***   - Legal guardians’ inability to provide consent to participate in the study. - A significant health condition which is known to affect lung function or imaging based on investigator discretion |

# Introduction

## Background

Almost all studies on the outcomes of pregnancy in females with cystic fibrosis (CF) were limited by small sample sizes and occurred prior to the widespread availability of CFTRm. Females with CF were found to have poorer outcomes, with a higher risk of pre-term birth, gestational diabetes and congenital abnormalities (1). Updated studies are now required to review if these prior conclusions are still valid given the increased health stability and improved survival conveyed by modulator therapy. Since the widespread introduction of CFTRm, there has been an associated dramatic increase in the number of pregnancies amongst females with CF (2,3), making this study important and timely.

Consistent with most novel drug therapies, females who were pregnant or actively trying to conceive were excluded from CFTRm clinical trials, meaning that all data on these medications during pregnancy has been gathered from retrospective reviews and case studies. Without robust evidence, decisions around continuing or ceasing CFTRm prior to or during pregnancy are challenging, particularly as CFTRm remain unlicensed for use in pregnancy. Despite this approximately 90% of females with CF elect to continue CFTRm during pregnancy (4). There have been reports of health decline in females with CF who have stopped CFTRm therapy during pregnancy (5), however this must be weighed against the potential for, as yet unknown consequences to the developing fetus.

Data from animal models has shown that all components of CFTRm drugs (most commonly used: elexacaftor, ivacaftor and tezacaftor (ETI) (6)) cross the placenta and thus result in a degree of fetal exposure. Although no specific therapeutic drug monitoring is available, studies did show that ETI levels have been found to be comparable in maternal, cord and infant blood, suggesting a therapeutic concentration(7). Low levels of ETI were also found in breastmilk.

Due to the rapidly changing landscape of CF health, there are no consensus guidelines currently for what, if any, clinical follow up or investigations those children born to females with CF should have. Suggestions from limited data concentrate on ophthalmological reviews for potential cataracts, and regular liver function testing in infants with continued drug exposure via breast feeding (7), but these are yet to become standardised or adopted into common practice.

An additional concern is the reported cases of infants with CF who had a delayed diagnosis due to a false negative newborn screen (9). This has occurred as a result of sufficient in utero absorption of ETI as to result in a negative screening result, due to correction of their CFTR function. These cases are rare but have serious health implications for the child who would have a delay in appropriate care and intervention at a key young age.

Further research is required to ensure all these infants are correctly managed. The US-based multicentre, prospective Maternal and Fetal Outcomes in the Era of Modulators (MAYFLOWERS) study (10) is investigating the association between maternal CF-health parameters and CFTRm-use but does not include a longitudinal assessment of the ‘obligate CFTR-heterozygote’ offspring. This study aims to start filling this gap in knowledge by providing high quality data, which will support further investigation and ultimately the production of guidelines on follow up practices for these infants.

## Pre-clinical and clinical data

### Pre-clinical data

ETI crosses the placenta and is detectable in breast milk, so will be encountered by the fetus in utero during critical organogenesis and by the infant early in postnatal development. Despite the small sample size in the Collins et al study (7) evidence of placental transfer of ETI suggests that the already established risks of ETI-administration in children such as hepatotoxicity, cataracts and hypertension should be considered.

Ivacaftor, a component of ETI, was first linked to cataracts in a pre-clinical study in juvenile rats (11). Rat pups which were dosed from postnatal days 7 – 35 developed cataracts at doses as low as 0.1X the maximum recommended human doses. However, a more recent animal study (12) which investigated CF-ferrets exposed to ivacaftor both in utero and throughout adulthood, did not show any lens abnormalities.

A 2024 study (13) investigated the tissue distribution pattern in rat fetuses exposed to maternally administered ETI. Fetal rats were exposed to seven days of maternally ingested oral treatment from day 12 to 19 of embryonic life. The fetal tissue sample was dissected at embryonic day 19 with analysis calculating an average entry ratio (based on tissue/plasma concentration) exceeding 100% in all tissues indicating accumulation of the drug. This was most significant in the liver at 200%, but levels in the lung, muscle, and small intestine were also approximately 100%.

In the same study Li et al. (13) also reported measurable ETI-accumulation in the embryonic rat-brain tissue at approximately 50% maternal rat-serum concentration, and although lower than other tissues, this confirms the ability of CFTRm components to cross the blood-brain barrier in this CF-animal model. Brain/plasma ratios in adult rats were significantly lower compared to the rat-pups suggesting the blood brain barrier’s greater ability to limit transfer reserved for older groups. In a recent case series (14) this possibility was raised when three children with CF developed raised intracranial pressure while on ETI, although causation could not be inferred and shifts in fat soluble vitamins could also be implicated.

The effect on the developing lung has also been raised. Although from a non-human in vitro model system, Hullier et al (15) reported that ETI, applied during the pseudo glandular stage of murine lung development, adversely affected lung branching and led to the formation of abnormal bronchial terminal dilatations. Data of this nature have not been replicated in in vivo animal or human studies.

### Clinical data

Existing studies of females with CF showed a degree of subfertility (15) when compared to the general population, thought to be multifactorial. Those females with CF who did get pregnant were found overall to have an increased rate of premature birth, lower birth weight infants, and higher risk of obstetric complications such as gestational diabetes (16). Significant bacterial pathogens were also implicated in the poorer longer-term outcomes, with a case series showing that three out of four women who died shortly following pregnancy were chronically infected with *Burkholderia cenocepacia* (18).

More recent studies have also looked at the implications of continuing/ceasing CFTRm in pregnancy. This has shown that those women who do stop will often restart due to significant deterioration in their own physical health (19)(5). Further studies have also shown that whilst the ante-partum period does not confer significant health risks, the first year of parenthood has resulted in increased risk of pulmonary exacerbations and overall health decline (20).

Whilst there are no known definitive side effects of CFTRm in infants exposed in utero, it is already recommended that those children with CF who are treated with ivacaftor or an ivacaftor containing compound should have yearly ophthalmology screening in case of cataract formation (11). It is unknown if in utero or lactation exposure to infants is equivalent to these directly administered therapeutic doses but raises questions already being explored.

Jain et al. (21) reported a case series in which 3 of 23 human infants born to mothers with CF taking ETI during pregnancy were diagnosed with bilateral congenital cataracts. The infants did not have any alternative explanation or risk factors for cataract formation. However, these cataracts were small, considered visually insignificant and were detected not on routine newborn eye checks using the red reflex, but at specialist paediatric ophthalmoscopy screening. The frequency of such subclinical changes in the general population is unknown. The current median prevalence of childhood cataract in the general population is 1 per 10,000 (22) with the sample size currently too small to make a comparison in an ETI exposed group.

Research into the associations with hepatotoxicity from in utero exposure of CFTRm is limited and in humans based solely on case reports. An infant from the Collins et al study (7) was reported to have a rise in ALT peaking at 65 U/L (upper limit normal 30 U/L) and self-resolving by day 158 with no alternative cause found. Another case report (23) with in utero exposure to lumacaftor/ivacaftor showed a raised AST (113 U/L) at day 29 in an infant which self-resolved after increasing the amount of formula milk relative to breast milk. Further case reports suggest mildly elevated liver enzymes in infants exposed to CFTRm via lactation (8,24), but again the variance within the general population is not well known, and there are many other causes of transient alterations in these enzymes.

In April 2021 the first known case of an infant with CF born to a mother on ETI throughout pregnancy was reported (7). Her 20-week antenatal obstetric ultrasound scan had showed echogenic bowel raising the suspicion of CF, although this finding resolved on later scans. The offspring was born at full-term, in good condition, with a normal newborn examination. The newborn screening IRT on day 2 was below the threshold for further genetic testing. However, due to the known paternal CF carrier status, the CF centre requested CFTR testing in the infant which confirmed a CF-affected infant homozygous for F508del. Faecal elastase was within normal limits, unusual for this mutation and further supporting the transplacental passage of ETI. Sweat chloride was also lower than expected at 5 weeks with levels of 60 mmol/L and 67 mmol/L with the infant breast feeding at the time.

A 2023 retrospective study(25) compared IRT levels between different groups of infants born in Indiana between 2020 - 2022. This included infants with IRT measurements below the cut-off levels for further testing, those diagnosed with CF, CF-carriers, and ETI-exposed infants (of which none were known to have CF). 191,493 infants were included, with 51 having CF, 489 carriers of a CFTR mutation, and 19 ETI-exposed. CF carriers not exposed to ETI had a mean IRT of 66.9 ng/ml while ETI exposed carriers had a statistically significant lower mean of 21.6 ng/ml (p <0.001). The mean for those with CF was 191.6ng/ml. Interestingly, the mean for those with an IRT below the threshold for testing, likely mainly non-carriers, was 25.4 ng/ml which was not significantly different to the ETI-exposed group (p=0.41). These findings suggest significantly lower levels of IRT in those exposed to ETI risking false negative results.

## Study Rationale and risk/benefit analysis

### Study Rationale:

There has been an increase in pregnancy rates in females with CF since the availability of CFTRm, and ETI in particular. This has created an opportunity for research into the experiences and management of females with CF during and immediately following pregnancy, in order to update guidelines and improve CF specific antenatal and postnatal management. This also extends to a better understanding of the impact on the females with CF of breastfeeding, something which has greater implications in CF due to its high metabolic demand. It is also important to use this opportunity to better understand how CFTRm, in particular ETI, impact the health of females with CF during pregnancy and how it may influence obstetric and neonatal outcomes.

This increase in pregnancy rates has directly resulted in an increase in infants exposed to CFTRm in utero or *via* breast milk. There is currently limited data on the health and developmental outcomes of these offspring. This includes the possibility of CFTRm exposure resulting in false negative CF-screening tests. This study aims to provide high quality data to support the creation of evidence-based guidelines on how best to manage this cohort of infants in the short and medium term.

The prioritisation of the need for this research is strongly endorsed by the receipt of a highly competitive CF Trust funded Strategic Research Centre grant following an international specialist peer-review process.

### Risks:

- Minimal pain associated with heel/finger prick blood tests/adult venepuncture.
- Sweat chloride tests can cause a tingling sensation on the skin but should be painless. On rare occasions a small burn can be caused.
- Lung MRI is a safe and painless investigation which does not involve any radiation. However:
  - it may scare some children due to loud noises or the tight spaces and the need to remain still.
  - it could be uncomfortable in later stages of pregnancy due to a need to lie supine.
  - it is also important to ensure that participants have no metal implants unsuitable for MRI.
- CT scans (‘Mama’ sub-study only) carry an additional risk of radiation. Some of the total radiation dose required by the study may be additional to routine clinical care for some participants. The risk of developing cancer as a consequence of taking part in this study is estimated as 0.03%.
- Qualitative interviews have the potential to cause distress, particularly around the recollection of traumatic or emotional events.
- Incidental findings of unclear significance discovered on investigations.
- Inconvenience of travel and visit burden to attend visits.
- Risk of communicable infections from attending visits in a hospital (see section 4.4)

### Benefits:

- Increased surveillance of possible CFTRm related sequalae allowing for appropriate management to be implemented if required.
- Contributing to data to support clinical guidelines on how best to manage and monitor infants exposed to CFTRm in utero or via lactation.
- Contributing to data to support how best to manage future pregnancies in females with CF.
- Increase understanding of the health impact of pregnancy and parenthood on females with CF to improve pre-conception counselling and post-pregnancy management and service delivery.

## Management of potential study risks

- Investigations carried out by qualified and trained health professionals.
- Public and patient involvement in design of study.
- Study based in a centre experienced in research.
- Infection control measures to avoid any cross infections with other patients. This will include the use of a private room for all visits and following local SOP for cross-infection control in CF outpatient clinic settings.
- Aligning maternal visits with standard care clinic visits to reduce study burden.
- Aligning infant visits with the timing of maternal visits.
- Working closely with parents to ensure comfort of the infant – for instance allowing breast feeding during heel prick blood test.
- Interviews are generally very low risk, although they often invite participants to explore sensitive or emotive topics (26). We will make it clear to participants that they can decline to answer specific questions or end the interview should they choose to do so. If it is clear to the interviewer that a participant finds particular questions or themes distressing, we will remind them that they can stop at any time. All participants will be provided with contact details of their clinical care team in case they have questions or would like to explore issues further after the interview. If the interviewer or analyser has concerns about a participant based on the content of the interviews, appropriate referral pathways will be followed.
- Incidental findings will be discussed with the appropriate clinician to provide context and a management plan.

# Study Aims and Objectives

## Primary aims

1. **Sub-study ‘Mama’**: To describe the impact of pregnancy and the first 12-24 months of parenthood in females with cystic fibrosis on their physical and psychological health.
2. **Sub-study ‘Mini’ & ‘Midi’:** To collect clinical data that will enable assessment of health outcomes in offspring of parents with CF in the short (up to 24 months) and medium-term (aged three-to-six years)

## Primary Objectives

1. **Sub-study ‘Mama’**
   1. To record the obstetric and neonatal outcomes of females with CF.
   2. To record CF-related health outcomes among females with CF including pulmonary (e.g. ppFEV_1_) and nutritional status (e.g. BMI) during pregnancy and early parenthood (up to 24 months).
   3. To record the subjective experiences of family planning, pregnancy and early parenthood in females with CF.
   4. To describe the impact of diabetes (pre-existing and gestational) on obstetric, neonatal and CF related outcomes.
   5. To record sweat chloride results during pregnancy and/or lactation among females with CF in relation to CFTR modulator therapy.
2. **Sub-studies ‘Mini’ and ‘Midi’**
   1. To collate clinically obtained serum liver enzymes levels and to record the frequency of congenital abnormalities (including ophthalmic cataract screening), in children born to a parent with CF (females or males with CF).
   2. To document cases of ‘missed’ CF-diagnoses due to false negative immunoreactive trypsinogen (IRT) results in children of females with CF in relation to transplacental and/or lactational exposure to CFTR modulators.
   3. To record lung function using lung clearance index (LCI) for young children (aged three-to-six years) born to a parent with CF.

## Exploratory Objectives

1. **Sub-study ‘Mama’**
   1. To provide biological samples to facilitate validation of a measurable assay for the individual components of CFTR modulators in maternal blood, and breastmilk.
   2. To record maternal serum and urine hormonal and micro-nutrient changes through pregnancy.
   3. To review lower respiratory tract microbiology in relation to pulmonary status (e.g. pulmonary exacerbation frequency and haemoptysis) among females with CF during pregnancy and early parenthood (up to 24 months).
   4. To identify changes in maternal bronchial artery size during pregnancy with antenatal radiological imaging (MRI) and explore rates of haemoptysis in pregnant and post-partum females with CF.
   5. To document fetal radiological findings in relation to embryological ophthalmic and pulmonary development.
2. **Sub-studies ‘Mini’ and ‘Midi’**
   1. To record blood pressure and cranial ultrasound measurements in children born to a parent with CF.
   2. To record routine neonatal health outcomes, offspring growth and developmental milestones in children born to a parent with CF.
   3. To record lung MRI results in young children (aged three-to-six years) born to a parent with CF.
   4. To provide biological samples to facilitate validation of a measurable assay for the individual components of CFTRm in the blood of babies exposed to CFTRm in utero and/or via lactation.

# Study design

## Overall design

This is a non-commercial, prospective, observational, mixed-methods study comprising three linked sub-studies, entitled **MATRIARCH_CF:** **‘Mama’, ‘Mini’, and ‘Midi’.**

A single, over-arching study protocol is warranted due to a continuation of participants, themes and data-sharing pregnant subjects (‘Mama’) and subsequent in utero environment in their offspring (‘Mini’ and ‘Midi’).

As an observational study, all the assessments described below will be optional, and participants will not be excluded from the study if they wish to decline specific investigations.

***Sub-study 1 – ‘Mama’ (pre-conception, pregnancy and parenthood)***

‘Mama’ is a sub-study of pre-conception, pregnancy and parenthood in adult females with CF. This is a prospective, mixed-methods, observational cohort study, collecting data generated in a clinical setting.

Participants will be recruited from the Royal Brompton Hospital (RBH) CF Reproductive and Maternal Health Service, up to 12 months prior to their having confirmed a planned date for actively trying to conceive, with follow-up until two years post-partum.

There will be up to a maximum of eight in-person study visits. These will occur:

- once up to 12 months pre-conception,
- once per trimester of pregnancy (X3), and
- at defined intervals post-partum.

All in-person visits will involve blood tests, lower respiratory tract samples, urine samples, lung function, health and quality of life questionnaires, in addition to taking biological samples for CFTRm assay (blood and breastmilk) and sweat chloride measurements.

The pre-conception, third trimester and 12-month post-partum in-person study visits will include radiological imaging which involves a paired synchronous lung CT and MRI when not pregnant or breastfeeding (or only MRI if visit occurs during pregnancy/lactation). In the third trimester this will also encompass a fetal MRI.

Standardised oral glucose tolerance tests will occur in the second and third trimester visits if clinically indicated (standard of care).

Semi-structured video interviews will be recorded for thematic analysis and will occur:

- once during pre-conception
- once ante-partum, and
- once post-partum.

***Sub-study 2 –’Mini’ (Offspring 0 - 24 months)***

The ‘Mini’ sub-study is a prospective, observational cohort study of children who have at least one biological parent with a diagnosis of CF. It is expected that many, but not all, of these infants will be those born to females participating in the ‘Mama’ sub-study. Any person with CF who is identified as having recently become or is due to become a parent through routine clinical visits in the RBH Adult CF Centre will also be eligible to enrol their offspring, up to 12 months of age.

Study visits for offspring exposed to CFTRm will follow the local clinical SOP (attached as a separate document) and will coincide with maternal visits in order to reduce study-burden. Tests proposed which will be additional to current clinical SOP are highlighted within this document (see table 2, section 9.4.1). All visits for those offspring *not* exposed to CFTRm, are solely for research purposes.

There will be a maximum of four in-person study visits for offspring participants within the first two years of life. The study visit review will be conducted by a paediatrician and dependent on CFTRm exposure status, will include:

- review of birth details,
- medical history (including ophthalmoscopy screening result),
- clinical laboratory tests (including liver function tests, sweat chloride, and faecal elastase),
- and physical examination.

Further investigations, not currently within our standards of practice, will include the addition of:

- Cranial ultrasound at the first visit,
- Serum CFTRm-assay levels,
- Serum samples for storage for further explorative testing if new research highlights further areas of interest.

***Sub-study 3 –’Midi’ (Offspring 3 – 6-year-olds)***

The ‘Midi’ sub-study is a cross-sectional, cohort sub-study of children aged three-to-six years who have at least one biological parent with a diagnosis of CF. Participants from ‘Mini’ will be eligible to progress into the ‘Midi’ protocol once the appropriate age has been reached. Participants will be enrolled via their parents who will be people with CF identified as under the care of the Royal Brompton Hospital Adult CF Service.

Participants who have been both exposed, and not exposed, to CFTRm will be invited to undergo the same investigations. The investigations are not invasive.

All participants aged three-to-six years will have a visit including the following, conducted by a paediatrician.

- review of birth details,
- medical history,
- neurodevelopmental milestone assessments,
- a multiple breath wash-out test to calculate lung clearance index (LCI),
- a sweat chloride test,
- physical examination,
- oxygen enhanced MRI (only performed in participants aged over five; those enrolled under the age of five will have a second visit once above five years of age as shown in section 6.2)

## Schematic of study design

Video recorded interview

Video recorded interview

Video recorded interview

MAMA

MIDI

MINI

# Eligibility criteria

## Inclusion criteria

***Inclusion criteria – ‘Mama’ sub-study:***

- Written informed consent obtained from participant.
- Under the care of Royal Brompton Hospital CF Reproductive and Maternal Health Service
- Age 16 years or above at time of recruitment.
- Confirmed diagnosis of CF.
- Planning a pregnancy or pregnant at time of recruitment.
- Ability to adhere to the required visits and investigations.

***Inclusion criteria – ‘Mini’ sub-study:***

- Written informed consent obtained from participant’s legal guardian.
- Infants who have a biological parent with a confirmed diagnosis of CF under the care of Royal Brompton Hospital Adult CF Service.
- Less than 12 months of age at first visit.
- Ability to adhere to the required visits and investigations.

***Inclusion criteria – ‘Midi’ sub-study:***

- Written informed consent obtained from participant’s legal guardian.
- Children who have a biological parent with a confirmed diagnosis of CF who is under the care of Royal Brompton Hospital Adult CF Service.
- Age three to six years at time of visit.
- Ability to adhere to the required visits and investigations.

## Exclusion criteria

***Exclusion criteria – ‘Mama’ sub-study:***

- Any significant health condition which would cause inability to comply with protocol based on investigator discretion.
- History of lung transplantation

***Exclusion criteria – ‘Mini’ sub-study:***

- Legal guardians’ inability to provide consent to participate in the study.
- Mother of infant has significant comorbidities unrelated to CF which could affect infant outcomes based on investigator discretion.
- A significant comorbidity in the infant unrelated to CFTR modulator exposure which could affect their outcomes, based on investigator discretion.

***Exclusion criteria – ‘Midi’ sub-study:***

- Legal guardians’ inability to provide consent to participate in the study.
- A significant health condition which is known to affect lung function or imaging based on investigator discretion.

# Recruitment process

Participant recruitment will only commence once the study team has ensured that the following approval/essential documents are in place:

1. The main REC, and Health Research Authority (HRA) approval.

2. Final sponsorship and/or confirmation of Capacity and Capability (C&C).

3. Sponsor has conducted the trial initiation procedure.

4. Signed delegation of Duties and Signature Log is completed.

All subjects who wish to enter the study will be screened and consented by the Chief Investigator, or one of the qualified clinicians involved in the study as Clinical Co-investigator.

All pregnant females with CF who are looked after by the Royal Brompton Hospital (RBH) will be approached to assess suitability based on medical notes and interest in taking part in the study. The CI leads the RBH CF Reproductive and Maternal Health Service where potential participants will be identified and provided with study information verbally. After this discussion they will receive a Patient Information Sheet (PIS).

Infants will be identified through the above service, either from mothers who are already pregnant when recruitment opens or those who become pregnant and are expected to deliver within the study timeframes. Parents of infants recently born and below 12 months of age will also be approached. It is anticipated that many of the infants will be born to mothers enrolled in the ‘Mama’ sub-study, but this is not an inclusion criterion. Those infants whose biological father is a person with CF under the care of the RBH Adult CF Service will be identified through the general CF clinics, and fathers given the same PIS.

After the infant is born inclusion/exclusion criteria will be assessed by a paediatrician via phone or video call and if eligibility is met they will be consented to enter the study..

The RBH CF Reproductive and Maternal Health Service have a database of children born to people with CF under their care. This database will allow the study team to locate children who are or will be between three to six years for inclusion in the ‘Midi’ sub-study. Parents of these children will preferably be contacted during their routine clinic visits, with follow up information sent via email and/or phone calls. If there are no scheduled clinic visits upcoming, they will be approached via phone. Furthermore, offspring enrolled in the ‘Mini’ sub-study can also take part in the ‘Midi’ sub-study as/when they reach the appropriate age within the study period.

Should parents be interested in the study, they will receive a PIS for which they will have at least 24 hours to consider whether they want their child enrolled in the study.

# Study procedures and assessments

Section 9.1. and 9.2 describe the procedures for informed consent and screening respectively, which must be carried out before any further assessments for all three sub-studies.

A description of each subsequent sub-study assessment is then described in section 9.3 for ‘Mama’ and section 9.4 for ‘Mini’ and ‘Midi’, with an associated schedule of assessments table.

## Informed consent

Eligible participants (or parents of eligible participants as per ‘Mini/Midi’ sub-studies) will be given a participant information sheet, *via* hand or email, and have a minimum of 24 hours to consider the invitation. Consent to enter the study will be obtained after a full account has been provided of its nature, purpose, risks, burdens and potential benefits, and the participants or parents have had the opportunity to deliberate. If there follows agreement to participate, informed consent will be obtained prior to study entry. The Investigator or designee will explain that the participants are under no obligation to enter the study and that they can withdraw at any time, without having to give a reason.

The consent process will be completed electronically through the REDCap database, or in person if a clinical visit is planned. For participants consented electronically, they will be able to download a copy of their consent form for their own records, The signed e-consent form will be stored in the secure REDCap study archive. A copy will be uploaded to the patient’s electronic medical records. One printed copy will be filed in the Investigator Site File (ISF).

If participants are consented in person, the original signed consent form will be retained at the study site (one filed in the medical notes, and one filed in the ISF). A copy of the signed consent form will also be given to the participant (or parents)*.*Informed consent will be obtained by the Chief Investigator, Principal Investigator and/or a nominated deputy as recorded on Sponsor’s Delegation of Responsibilities Log. All individuals taking informed consent will have received training in Good Clinical Practice (GCP).

## Screening assessments

Subject to receipt of informed consent from participants/parents, the inclusion and exclusion criteria will again be reviewed with the participants/parents and by using their medical notes.

## ‘Mama’ sub-study assessments

### Summary flow chart of study assessments (Table 1)

Assessments that are performed in addition to clinical care are highlighted in grey.

| Study Procedure | Pre-conception | 1^st^ trimester | 2^nd^ trimester | 3^rd^ trimester | 4-8 weeks PP | 6 months PP | 12 months PP | 24 months PP |
| --- | --- | --- | --- | --- | --- | --- | --- | --- |
| Informed consent | X |  |  |  |  |  |  |  |
| Inclusion/exclusion criteria | X |  |  |  |  |  |  |  |
| Demographics | X |  |  |  |  |  |  |  |
| Sputum sample | X | X | X | X | X | X | X | X |
| Clinical Blood tests^2^ | X | X | X | X | X |  | X | X |
| Blood test for CFTRm assay | X | X | X | X | X | X | X | X |
| PAPP-A and HCG |  | X |  |  |  |  |  |  |
| Urine sample | X | X | X | X | X |  |  |  |
| Lung function | X | X | X | X | X | X | X | X |
| Sweat test | X | X | X | X | X |  |  |  |
| Lung CT^1^ | X |  |  |  |  | X | |  |
| Lung MRI^1^ | X |  |  | X |  | X | |  |
| Fetal MRI |  |  |  | X |  |  |  |  |
| EPDS^3^ |  |  |  | X | X |  |  |  |
| Questionnaires^4^ | X | X | X | X | X | X | X | X |
| Semi-structured interview | X |  | X | |  | X | |  |
| Breast milk sample for CFTRm assay^5^ |  |  |  |  | X | X | X |  |
| Key:  X: Planned completion date   1. Lung Imaging – CT will only be performed on non-lactating and non-pregnant participants. Post-partum CT and imaging will be paired, so either a CT and MRI done at the same visit if no longer breastfeeding, or a single MRI if still breastfeeding at 1 year 2. Clinical blood tests – Full blood count, renal profile, liver function, Vitamin A, D, E and K, CRP, OGTT if clinically relevant, HbA1c 3. EPDS – Edinburgh Postnatal Depression Score 4. Questionnaires to include CFQ-R, EQ-5D-5L, PHQ9 and GAD7 5. If breastfeeding whilst taking CFTRm | | | | | | | | |

### Baseline assessments

Screening/baseline assessments are done at the first study visit. There is no separate screening visit.

- Physical examination and history
  - Weight, blood pressure (BP), oxygen saturations, heart rate (HR).
  - Spirometry.
  - Medication list including CFTRm dosages.
  - Review of previous medical history/registry data (if previously consented to having data stored on the CF Trust Registry).
- Clinical tests
  - Sputum microbiology.
  - Sweat chloride.
  - Blood tests – renal function, full blood count, liver function, fat soluble vitamins, sample for CFTRm assay, storage samples of serum and urine (e.g. micro-nutrient analysis, hormonal profile, PAPP-A).
- Questionnaires
  - - CFQ-R
    - EQ-5D-5L
    - PHQ-9 and GAD 7
- Qualitative Experience
  - - Video recorded semi-structured interview.
- Imaging
  - - Paired lung CT/MRI (if enrolled when pregnant – MRI only).

### Medical History

For adult participants who are patients under the Royal Brompton CF service, permission to access past medical details from medical records (including from the UK CF Registry if participant has previously consented to this) will be requested to be used in analysis of the study results.

### Sweat test

A small electrical current is applied to the forearm, following which a collecting device is used to collect up to 0.5ml of sweat over a 20 - 30-minute period. This is done on both forearms in parallel. The sample will be sent and processed by our local laboratory.

### Blood tests

Venepuncture equipment will be used to take up to 20mls of venous blood. Clinical samples will be sent to the local laboratory for analysis for full blood count, liver function tests (ALT, ALP, AST, Gamma-GT, total bilirubin, and albumin), vitamin profile (vitamins A, D and E), renal function (urea, creatinine, eGFR, potassium and sodium). Tests will be analysed as per local lab procedures.

One sample will be designated for CFTRm assay validation and storage (a serum sample (ca. 200 microliters if singlicate) for qualitative and quantitative analysis of CFTRm and their major metabolites using LC-HRMS or LC-MS/MS).

A further sample will be for micronutrient analysis, PAPP-A and hormonal e.g. β-hCG analysis – this will be run as batches.

### Breastmilk sample

Participants will be asked to express up to 5ml of breastmilk which can be done at home or in a clinical setting at their preference. These will then be stored and used for validation of CFTRm assay.

### Qualitative Interviews

Qualitative interviews would take place via MS Teams with a member of the research team and would be recorded and transcribed using MS Teams software. We will make it clear to participants that they can decline to answer specific questions or end the interview should they choose to do so. If it is clear to the interviewer that a participant finds particular questions or themes distressing, we will remind them that they can stop at any time. We would anticipate the interviews taking between 30-60 minutes.

### Spirometry

This is usually performed during or prior to scheduled clinic visits using a handheld home spirometry device as part of standard care. The results are visible through the linked online application. Additional laboratory spirometry would not be required.

### Sputum samples

If the participant is able to produce a sputum sample this would be taken and processed for microbiology as per the clinical microbiology SOP. If unable to produce a sputum sample, then a cough swab or induced sputum (if not pregnant) would be an acceptable alternative (both regular clinical practice). Excess sputum not required for clinical evaluation would be stored and saved for potential further research analysis.

### Urine sample

Participants would be able to bring this sample from home in a universal container. The first urine of the day is preferred but this is not essential. A minimum volume of 5ml would be required.

### CT scan

A lung CT scan will take place in the first visit pre-conception with a second CT scan planned for 6-12 months post-partum if not breast feeding.

### Oxygen-enhanced Magnetic Resonance Imaging (OE-MRI) of lungs

Up to three oxygen enhanced lung MRIs are proposed:

- 1. prior to conception,
  2. during the third trimester, and
  3. six-months post-partum.

These MRIs will focus on the adult participant’s lungs to assess for any structural or functional changes.

Before the MRI is carried out, the study team will confirm that the participant does not have any contraindications to MRI using the local MRI safety checklist. If the participant is unable to tolerate the MRI for any reason, the procedure will not go ahead. Sedation is not an option for this study.

Headphones will be provided to support increased comfort in the scanning machine. All scans will be performed lying supine, but for the scan in the third trimester this will be a ‘supported supine’ position using pillow/wedges to adjust positioning and improve comfort.

The scan will take 20 – 40 minutes and will involve a short period of breathing 100% oxygen via a facemask. The study team is very experienced in performing oxygen enhanced lung MRI in adults and children and the broader research team has previously performed oxygen enhanced placental imaging via MRI during pregnancy. If any abnormalities are detected on the scan, they will be discussed with the clinical team. Participants will be consented about the risk of false positive or incidental findings. There is no associated radiation risk with MRI, and it can be safely performed in pregnancy.

### Fetal MRI

The fetal MRI will be undertaken at the same time as the maternal third trimester scan. The maternal images will be obtained first with fetal imaging obtained immediately after. There will be a focus on imaging the fetal eyes for lens opacities, and the lung for any changes in lung development. This will add a further 10 - 20 minutes to the MRI time. Depending on emerging research, images obtained may later be processed to review other areas of anatomy, development and function. Consent forms will include this option.

### Questionnaires

The questionnaires being used are validated study questionnaires, and some are therefore subject to copyright restrictions in terms of the forms distributed to participants. Some have a section for participant’s name. The original questionnaires will be kept in the patient’s medical notes as they have clinic relevance. The results will then be transcribed and stored anonymously on the research database under the participants research identifier.

## ‘Mini’ and ‘Midi’ sub-study assessments

### Summary flow chart of study assessments (Table 2 & 3)

‘Mini’ Sub-Study (Table 2)

A phone or video call will take place before the first in-person visit to review inclusion/exclusion criteria and will be repeated in person at the first visit.

There are no strict time-windows in which each visit must be completed, but preferably they should be as close as possible to the designated visit. If a participant is enrolled at an older age than 8 weeks (for example any age up to 12 months), all the procedures from Visit 1 should still take place at the first visit, with the schedule then proceeding as below.

| Study Procedure | Visit 1  4-8 weeks age | Visit 2  6 months of age | Visit 3  12 months of age | Visit 4  24 months of age |
| --- | --- | --- | --- | --- |
| **For all children enrolled** | | | | |
| Informed consent | X |  |  |  |
| Inclusion/exclusion criteria | X |  |  |  |
| Demographics | X |  |  |  |
| Baseline assessments^1^ | X |  |  |  |
| Medical History^2^ | X | X | X | X |
| Physical examination^3^ | X | X | X | X |
| Liver function blood tests^4^ | X | X | X^5^ | X^5^ |
| Sweat test^6^ | X | X^7^ |  |  |
| Faecal elastase^8^ | X | X^7^ |  |  |
| Cranial ultrasound^9^ | X |  |  |  |
| **Only for children exposed to CFTRm** | | | | |
| Document ophthalmological review^10^ | X |  | X^5^ | X^5^ |
| CFTRm assay^11^ | X | X | X^5^ | X^5^ |
| **Key**:  X: Planned completion date  Highlighted in grey: tests that are additional to standards of practice. Note that all tests for children not exposed to CFTRm are additional to standards of practice.  1. See section 9.4.2  2. See section 9.4.4.  3. See section 9.4.5.  4. See section 9.4.6  5. Only if breastfed and exposed to CFTR modulator in last 6 months  6. See section 9.4.8  7. If exposed to CFTRm via breast feeding at the 4 – 8 weeks visit to repeat at 6 months. If still exposed at 6 months repeat at 12 months and so on, as exposure to CFTRm via lactation could affect results  8. See section 9.4.9.  9. See section 9.4.10  10. See section 9.4.3  11. See section 9.4.7 | | | | |

‘Midi’ Sub-study (Table 3)

As for ‘Mini’, a phone or video call will take place before the first in-person visit to review inclusion/exclusion criteria and will be repeated in person at the first visit.

All tests for all participants in the ‘Midi’ study are additional to standards of practice.

| Study Procedure | Age 3 - <5 years at time of recruitment | | Age 5+ at time of recruitment |
| --- | --- | --- | --- |
|  | First visit | Second visit (once aged 5+) | Single visit |
| Informed consent | X |  | X |
| Inclusion/exclusion criteria | X |  | X |
| Demographics | X |  | X |
| Baseline assessments^1^ | X |  | X |
| Medical history^2^ | X | X | X |
| Physical examination^3^ | X | X | X |
| Sweat test^4^ | X |  | X |
| O2 enhanced Lung MRI^5^ |  | X | X |
| Lung clearance index^6^ | X | X | X |
| **Key**:  X: Planned completion date  1. See section 9.4.2  2. See section 9.4.4.  3. See section 9.4.5.  4. See section 9.4.8  5. See section 9.4.12  6. See section 9.4.11 | | | |

### Review of medical records (baseline assessment)

The review of the child’s and parental medical records as listed below should be completed using a combination of parental reporting and medical records.

For all participants the below information should be recorded:

- Gestation in weeks, singleton/multiple birth, mode of delivery, APGAR score (at 1/5/10 min), admission to SCBU/NICU (Yes/No, and indication)
- Newborn blood spot: immunoreactive trypsinogen (IRT) result
- Birth weight in kilogram and centile, head circumference in centimetres and centile.
- NIPE outcome (normal/abnormal; specify abnormality)
- 6-week GP examination result
- Maternal health:
  - - Age, comorbidities, medications, ante/perinatal complications
- Parental genetics (to be accessed via relevant medical notes after informed consent obtained from each parent):
  - CF genotype of parent with known diagnosis
  - CF genetic screening result of non-CF parent if performed.

For participants exposed to CFTRm during pregnancy and or via lactation, to document:

- CFTR genetics results
  - - Would only have been carried out if mother has CF and male partner is either a known carrier, has unknown CFTR genotype, or had only undergone a common variant screen.

### Documentation of ophthalmology review

For children exposed to CFTRm in utero or via lactation they will be referred to ophthalmology as per local standards of care. This review is to rule out lens opacities (cataracts). This ideally should happen in the first two months of life if *in utero* exposure and then yearly while exposed to CFTRm via breast feeding. Parents should be asked to bring the letter of the review to then document whether cataracts were observed or not.

### Medical history

For all participants document the below information:

- Any history suggestive of CF
  - - Number of chest infections, wet cough, constipation, greasy stools, faltering growth
- Feeding history (breast/bottle/mixed and estimated percentage of each)
- Achievement of age-appropriate developmental milestones
  - Specifically, assessing for achievement of milestones prior to limit ages for areas of gross motor, vision and fine motor, hearing, speech and language, and social behaviour (see details in appendix 2 based on Lissauer, T. and Clayden, G., 2015. *Illustrated textbook of paediatrics*. 4th ed. Edinburgh: Elsevier)(27)
- Medication history
- Has there been continued/new exposure to CFTR modulators:
  - - In-utero (No, Yes (timing and months of exposure))
    - Breast feeding while mother on CFTRm (No, Yes (timing and months of exposure)

### Physical examination

At all visits carry out the below examinations using local procedures:

- Weight in kilograms (and plot centile)
- Head circumference in centimetres (and plot centile) if below the age of two.
- Blood pressure in millimetres of mercury (and plot centile)
- Document any abnormalities detected in a general examination including the cardiovascular, respiratory, abdominal, and neurological systems.

### Liver function blood tests

A heel/toe prick device will be used to obtain around 1ml of capillary blood into an SST bottle and sent to the local laboratory for analysis for liver function tests (ALT, ALP, AST, Gamma-GT, total bilirubin, conjugated bilirubin, unconjugated bilirubin, and albumin). In circumstances where parents prefer venepuncture over a heel prick this will be an option. Tests will be analysed as per local lab procedures.

Three monthly liver function tests are our local standard of practice for infants with continued exposure to CFTRm in the first year. The blood test temporally closest to the study visit will be recorded. If any abnormalities are found, there may be a requirement for more regular testing. This will also be recorded.

For infants not exposed to CFTRm this test is for research purposes only, and as with all other tests optional.

### Blood test for CFTRm assay and serum save

During the same procedure as the liver function blood tests, around 200 microliters of blood will be collected for qualitative and quantitative analysis of CFTRm and their major metabolites using LC-HRMS or LC-MS/MS and storage. If there is remaining sample, it will be stored and frozen for potential future analysis of other tests if emerging research suggests this is necessary.

### Sweat chloride testing

A small electrical current is applied to the forearm, following which a collecting device is used to collect up to 0.5ml of sweat over a 20 - 30-minute period. This is done on both forearms in parallel. The sample will be sent and processed by our local laboratory.

### Faecal elastase

A stool sample will be collected by parents in a universal container and sent to the local laboratory for quantification of faecal elastase levels.

### Cranial ultrasound (‘Mini’ sub-study only)

A consultant paediatric radiologist will carry out a focussed cranial ultrasound scan at the first infant visit at 4 – 8 weeks of age. If not carried out in visit one, this can also be carried out at visit 2 (6 months of age). There will also be the opportunity for repeat scans dependent on initial findings, radiologist availability and parental preferences.

This purpose of this scan is to assess for changes indicative of raised intracranial pressure (which has been described as a possible side effect of childhood use of ETI). This will include measurements of the fluid spaces within the baby’s brain and of blood flow within the arterial system of the brain. The scan will take around five minutes.

There is no associated radiation risk with ultrasound. The scan is not uncomfortable for the baby with an ultrasound probe lightly placed on the anterior fontanelle. If needs be, the scan can be performed whilst the baby is cradled. If the baby is uncomfortable for other reasons and cannot easily be settled the scan will not proceed and can be rearranged. This scan also raises the possibility of incidental findings of unclear significance. These findings will be discussed as appropriate with a paediatrician to create a management plan.

### Lung clearance index (LCI) (‘Midi’ sub-study only)

A qualified RBH physiologist will use an Exhalyzer D to perform a multiple breath washout (MBW) test and provide a lung clearance index (LCI) report(28,29). LCI, the primary derivative from MBW, is a measure of the efficiency of gas mixing within the lungs and an indicator of ventilation inhomogeneity. It is a sensitive measure in detecting peripheral airway changes in children. The test requires relaxed tidal breathing while the child breathes in 100% oxygen for a few breaths. It is repeated to obtain three reliable trials, in which the mean result is taken as the LCI result. Overall, this should take around 20 – 30 minutes.

### Oxygen (O_2_)-enhanced Lung MRI (‘Midi’ sub-study only)

At the visit when the child is between 5 – 6 years of age an O_2_-enhanced MRI of the child’s lungs will be undertaken to assess for any structural or functional changes. The study team is experienced in performing these scans on children of this age. Before the MRI is performed, the study team will confirm that the child does not have any contraindications to MRI using the local MRI safety checklist. If the child is unable to tolerate the MRI for any reason, the procedure will not go ahead. Again, sedation is not an option on this study. Headphones with music and other calming measures will be provided to increase comfort in the scanning machine and a parent is encouraged to remain in the scanner room to keep the child calm. Information from the RBH clinical specialist ‘play team’ will be provided in advance to allow some pre-scan preparation.

The MRI scan will take 20 – 40 minutes and will involve a short period of breathing 100% oxygen via a facemask. If any abnormalities are detected on the scan, they will be discussed with the paediatricians on the study team and any appropriate further investigation or follow-up arranged. Families will be consented about the risk of false positive or incidental findings. There is no associated radiation risk with MRI scans.

# Definition of the End of Study

Completion of the last participant’s last visit.

# Discontinuation/withdrawal of participants

Participants are able to withdraw at any time point. They will be able to request their data are removed from the study. Data that has been anonymised and merged will not be able to be removed, including samples which have been stored for block analysis.

# Safety Reporting

## Definition

**Adverse Event (AE)** — any untoward medical occurrence in a patient or clinical study subject who is participating in a study.

**Serious Adverse Event (SAE)** – is defined as an untoward occurrence that:

- Results in death; or
- Is life-threatening (places the subject, in the view of the Investigator, at immediate risk of death)
- Requires hospitalization or prolongation of existing hospitalization (hospitalisation is defined as an inpatient admission, regardless of length of stay; even if it is a precautionary measure for observation; excluding hospitalisation for an elective procedure, for a pre-existing condition)
- Results in persistent or significant disability or incapacity (substantial disruption of one’s ability to conduct normal life functions)
- Consists of a congenital anomaly or birth defect (in offspring of subjects or their parents taking the study drug regardless of time of diagnosis)
- Is otherwise considered medically significant by the investigator.

Important medical events that may not be immediately life-threatening or result in death or hospitalisation but may jeopardise the subject or may require intervention to prevent one of the outcomes listed in the definition of serious will also be considered serious.

## Recording Adverse Events (AEs)

This study is an observational study with no treatment provided. The investigator will discuss with the participant whether there have been any occurrence of AEs. Only AEs deemed to be (possibly) related to the study procedures need to be strictly recorded in the medical records and CRF. These should be recorded in full including the duration (start and end dates), severity (Table 4), outcome, treatment provided, and relation to study procedures (Table 5), as well as if it meets the definition of an SAE.

Non-study procedure related AE’s can be recorded in the medical records if felt to be clinically significant. Any AEs felt to be related to CFTRm should be reported via the national Yellow Card Scheme.

**Table 4. AE Severity grading**

| Severity | Description |
| --- | --- |
| Mild | Asymptomatic or mild symptoms; clinical or diagnostic observations only; intervention not indicated |
| Moderate | Minimal, local or non-invasive intervention indicated; limiting daily activities. |
| Severe | Medically significant but not immediately life-threatening; hospitalization or prolongation of hospitalization indicated. Unable to perform daily activities. |
| Life-Threatening | Life-threatening consequences: urgent intervention indicated. |
| Death | Death related to AE. |

**Table 5. AE relationship to study procedure**

| Relationship | Description |
| --- | --- |
| Unrelated | There is no evidence of any causal relationship |
| Unlikely | There is little evidence to suggest there is a causal relationship (e.g. the event did not occur within a reasonable time after study procedure). There is another reasonable explanation for the event (e.g. the participant’s clinical condition, other concomitant treatment). |
| Possibly | There is some evidence to suggest a causal relationship (e.g. because the event occurs within a reasonable time after study procedure). However, the influence of other factors may have contributed to the event (e.g. the participant’s clinical condition, other concomitant treatments). |
| Probably | There is evidence to suggest a causal relationship and the influence of other factors is unlikely. |
| Almost certainly | There is clear evidence to suggest a causal relationship and other possible contributing factors can be ruled out. |

## Assessment of SAEs

Classification and causality of Adverse Events (AEs) will be conducted by sub-investigators and reviewed by the CI. The CI cannot downgrade the initial classification and if there is disagreement which cannot be resolved during formal discussion then the assessment of the sub-investigators will be accepted. The CI, can however, upgrade the seriousness of an event without consultation with the sub-investigator.

Only SAEs felt to be related to study procedures will be reported as described in section 12.5.

## Expected AEs

No intervention or treatment is being provided. We also do not anticipate any study procedure related AE or SAE’s, but if these do occur, they will be recorded in the hospital notes and case report form (CRF).

In adult patients' temporary health deterioration and possible admission to hospital is expected and will be recorded as part of the study.

In the ‘Mini’ and ‘Midi’ study AEs related to CFTR modulators such as liver dysfunction or cataracts will be reported through the yellow card scheme, and participants will be medically managed by the paediatricians in the team. This may lead to extra clinical visits and further testing where clinically required.

## Reporting of SAEs to the sponsor and the REC

The CI and their research team at RBHH are responsible for reporting study procedure related SAEs to the Research Office immediately and/or within 24 hours of becoming aware of the event in accordance with the process outlined below.

A study procedure related SAE occurring to a research participant will be reported to the Research Ethics Committee (REC) that gave a favorable opinion of the study, the study Sponsor (RBHH Research Office) and the local R&D Office where in the opinion of the CI/PI the event was:

- **‘Related’**: that is, it resulted from administration of any of the study procedures; and
- **‘Unexpected’**: that is, the type of event is not listed in the protocol as an expected occurrence.

Reports of related and unexpected SAEs will be submitted to the REC within 15 days of the CI/PI becoming aware of the event; using the SAE reporting form for non-CTIMPs published [on the HRA website and entitled non-CTIMP safety report to REC](file:///C:\Users\ij476\AppData\Local\Microsoft\Windows\INetCache\06_Drafts\03_RO%20Guidance%20Documents\on%20the%20HRA%20website%20and%20entitled%20non-CTIMP%20safety%20report%20to%20REC). The form should be completed in typescript and signed by the Chief Investigator (CI) prior to submission to the REC.

All SAEs that are to be reported to the REC should also be forwarded to the Research Office in parallel and must be recorded, signed and dated by the Investigator at site. Research Office accepts study specific SAE forms, HRA SAE Form or RBHH template SAE Reporting Form available [here](http://www.rbht.nhs.uk/research/for-researchers/setting-up-and-managing-a-project/policies/templates/).

Information can be submitted to the Research Office in electronic format:

- E-mail: [safetyreporting@rbht.nhs.uk](mailto:safetyreporting@rbht.nhs.uk).

Following submission by the CI, the coordinator of the main REC will acknowledge receipt of safety reports within 30 days. It is the responsibility of the CI and his/her research team to send a copy of the SAE notification and acknowledgement receipt to the Research Office.

The study team also has the responsibility to report SAEs occurring in a certain period (28 days) after a patient completes the study. Any SAEs reported to the Investigators during this phase must be documented in the patient’s medical notes and submitted *via* an SAE reporting form.

## The type and duration of Follow up

This study does not include any intervention. Therefore, no follow up requirement for AE/SAEs related to the study are anticipated for the participants. However, the study team includes paediatric and adult trained doctors who will be able to support participants to ensure appropriate management and follow up is in place in the RBH or elsewhere if needed for any other medical issues that arise. This may include the need for extra clinic appointments, investigations and treatments. These will be documented in the final analysis.

## Annual Progress Reports (APRs)

The Chief Investigator will prepare the APR for the study. It will be sent to the REC by the CI within 30 days of the anniversary date on which the favourable opinion was given by the REC, and annually until the study is declared ended.

## Reporting Urgent Safety Measures

The Sponsor and/or the Investigator may take appropriate urgent safety measures in order to protect the subjects of a clinical study against any immediate hazard to their health or safety. If safety measures are taken, REC approval is not required before the measure is taken.

The Investigator will immediately and in any event no later than 3 days from the date the measures are taken, give written notice to the REC and the study Sponsor of the measures taken and the circumstances giving rise to those measures.

In order to prevent any delays in the reporting timelines the Sponsor has delegated this responsibility to the CI/PI. Therefore, the CI/PI must report any urgent safety measures to the REC directly, and in parallel to the Sponsor. The REC coordinator will acknowledge receipt of urgent safety measures within 30 days.

## Notification of Serious Breaches of GCP and/or the protocol

To ensure compliance with the REC favourable ethical opinion, the sponsor should report to the REC any serious breaches of the protocol or of the principles of Good Clinical Practice. A “serious breach” is defined as a breach of the protocol or, of the principles of Good Clinical Practice which is likely to affect to a significant degree the safety or physical or mental integrity of the study participants, or the scientific value of the research. There is no requirement to notify minor breaches of GCP or the protocol.

It is the responsibility of the CI to ensure that any non-compliance is reported to the Research Office, to allow appropriate investigation.

Reports of non-compliance should give details of the issues that have occurred, the location, who was involved, the outcome and any information given to participants. In the instance of reporting non-compliance as a serious breach, the Research Office will work with the study team to generate an explanation to ensure the REC is informed of further action the sponsor plans to take.

# Data management and quality assurance

## Confidentiality

All data will be handled in accordance with the Data Protection Act 1998, NHS Caldecott Principles, The Research Governance Framework for Health and Social Care, 2nd Edition (2005), and the condition of the main REC approval.

The Case Report Forms (CRFs) will not bear the subject’s name or other personal identifiable data. A study Identification Number (ID) will be used for identification. This number will not include any personal information. Only the unique ID will be used on any study-related information collected. Information that contains a participant's identity will remain only with the study team.

No information or records that disclose a participant's identity will be released without consent unless required by law.

## Data collection tool

To support continuity of care data collected will be recorded on the subject's secure password protected Royal Brompton Hospital electronic medical notes. Case Report Forms (CRF) have been designed to record specific information required from the medical notes for study data analysis. These CRFs have been designed by the CI and Sub-I's and the final version will be approved by the Sponsor. CRF’s will be electronic and held on REDCap, a secure password protected database.

It is the Investigator’s responsibility to ensure the accuracy of all data entered and recorded in the CRFs. The Delegation of Responsibilities Log will identify all trial personnel responsible for data collection, entry, handling and managing the database.

## Data handling and analysis

REDCap is a secure password protected web-based database which will be used for data entry, storage and analysis. Disaster recovery of this data is included on these servers, with files regularly backed up.

The CRF saved on REDCap will be designed to ensure all the required data is collected. The sub-investigators and other delegated staff will be responsible for data entry. Staff will be trained to ensure consistency in data input and adherence to the protocol.

All samples transferred to other sites will only be coded with data ID rather than patient identifiable data. Results of this will be processed in batches and the results returned to researchers via a secure transfer.

Data analysis will be done when final study data is available as no formal interim analysis is planned, and prior to this a statistical analysis plan will be written, taking into account the numbers recruited.

# Archiving arrangements

The study documents (including the Trial Master File (TMF), Case Report Forms (CRFs), Informed Consent Forms along with the trial database) will be kept for a minimum of five years. They will be stored in locked offices within the Royal Brompton and Harefield NHS Foundation Trust site for the duration of the study. The Chief Investigator is responsible for the secure archiving of study documents. The study database will also be kept electronically on the Trust computer network, for a minimum of five years.

The approved repository for longer retention of local materials for studies that involve RBHH patients is OASIS Group UK. The study documentation will be prepared for archiving by the study team in line with the Research Office Archiving SOP and the transfer will be arranged by the Research Office.

# Statistical design

## Statistical input in study design

All three sub-studies described in this protocol are observational in design. Descriptive analyses will be used to report the data. Within this context no complex statistical analyses are anticipated which require statistician input in the design phase and a statistical analysis plan will be written prior to final analysis.

## Endpoints

### Primary endpoints

As an observational study, a combination of multiple endpoints will be monitored to identify areas of interest that may warrant and benefit from future investigation in larger studies.

Below are the endpoints which will be explored based on the currently available literature and research in the field.

*‘Mama’ sub-study*

1. Change in percent predicted FEV_1_ (ppFEV_1_) from pre-conception/baseline to end of pregnancy, and at 12- and 24-months post-partum.
2. Incidence of CF-related pulmonary complications during pregnancy.
3. Incidence of premature delivery (defined as birth <37 weeks gestation).

*Combined ‘Mini’ and ‘Midi’ sub-study*

1. The number of participants with:
2. Liver dysfunction (ALT/AST/GGT/bilirubin above upper limit of normal). This is prospectively obtained in the ‘Mini’ sub-study, and retrospectively reviewed from medical records in the ‘Midi’ sub-study.
3. Presence of congenital abnormalities including cataracts (based on medical history and examination)
4. The number of participants with normal IRT subsequently diagnosed with CF (based on a combination of CF genetic tests, sweat chloride, and faecal elastase where appropriate)
5. The number of participants with an abnormal lung clearance index (LCI) (‘Midi’ only)

### Exploratory endpoints

*‘Mama’ sub-study*

Quantitative endpoints are:

1. Obstetric-specific:
   1. Caesarean section rate
   2. Incidence of medically initiated delivery for pulmonary indication
   3. Incidence of intrahepatic cholestasis
   4. Incidence of pregnancy induced hypertension.
   5. Incidence of pre-eclampsia
   6. Incidence of gestational diabetes
   7. Incidence of large or small for gestational age infants
   8. Incidence of postnatal anxiety/depression (as noted by EPNDS or patient-reported following clinical assessment by other healthcare providers)
   9. Obstetric outcomes according to chronic airways pathogen colonisation history
   10. Obstetric outcomes according to micro-nutritional or early pregnancy hormonal status.
   11. Obstetric outcomes according to diabetes status and therapy (CF diabetes, gestation diabetes, no diabetes)
2. CF-specific:
   1. Incidence of pulmonary exacerbations during pregnancy and within first 24 months requiring oral and/or IV antibiotics.
   2. Variability in lung function during and post-pregnancy as shown by OE-MRI
   3. Sweat chloride levels during pregnancy, and variability per trimester.
   4. Change in weight from pre-conception until pre-delivery.
   5. Change in CFQ-R, EQ-5D-5L, PHQ9 and GAD7 scores.
   6. Bronchial artery size assessment (by MRI) at baseline/pre-conception, antenatal and post-partum (6-12 months).
   7. Change in haemoptysis frequency from baseline, and during pregnancy (as per number episodes per person-years pregnant), and post-partum.
   8. Change in weight according to breast-feeding status in first 24 months post-partum.
   9. Validation of CFTRm assays in serum and breastmilk.

Qualitative endpoints:

1. Thematic analysis of experiences of females with CF throughout:
   1. Family planning,
   2. Pregnancy and childbirth,
   3. Early parenthood.

*Combined ‘Mini’ and ‘Midi’ sub-study*

Exploratory endpoints are:

1. Number of participants with below neonatal birth outcomes:
   1. Prematurity (defined as being born < 37 weeks' gestation)
   2. Low birth weight < 2^nd^ centile
   3. Special care or neonatal intensive care unit admission
2. Frequency of raised blood pressure (>95^th^ centile for age, sex and height on two separate readings)
3. Frequency of faltering growth (definition as per NICE guidelines [NG75])
4. Frequency of delayed development past limit ages in gross motor, vision and fine motor, hearing, speech and language, and social domains. (see appendix 2)
5. Number of participants with raised intracranial pressure diagnosed on cranial ultrasound (‘Mini’ only)
6. Validation of a CFTRm assay in serum (‘Mini’ only)
7. Abnormal lung branching on lung MRI. (‘Midi’ only)

## Sample size and recruitment

### Sample size calculation

This is an observational study designed to inform potential future studies. Recruitment will be pragmatic and designed to maximise participant numbers, without a pre-determined sample size from within a limited pool.

There are currently insufficient studies to provide data for power calculations. This study will provide pilot data to help inform whether further studies are warranted and provide data to power future studies.

### Planned recruitment rate

***‘Mama’ sub-study***

The aim of recruitment will be to maximise number of participants in the study. Approximately 15 women complete a pregnancy (give birth to a live infant) a year at the Royal Brompton Hospital CF Maternal Health Service, and patient involvement workshops indicate approximately two-thirds would be inclined to enrol in the trial. Due to the follow up duration we would expect to be able to recruit 20-25 participants.

Participants will be eligible to enrol at any stage in their pregnancy and will be consented for retrospective access to clinical data collected prior to enrolment that is included in the study.

*‘****Mini’ sub-study***

Mirroring the Mama sub-study, with most infants expected to be enrolled via this route, this will provide an overall pool of 15 babies per year. With two-thirds expected to enrol this will lead to around 10 infants per year. Children already born and under the age of 12 months at first visit can also be included which will add an estimated five further infants to the study. Overall, the expected recruitment over 3-year period will be 25 – 35 infants.

***‘Midi’ sub-study***

There is an existing cohort of around 30 infants born to a parent with CF aged 3 – 6. Anticipating around a third of these will enrol to the study should lead to around 10 participants in the first year of the study.

Infants newly turning three during the duration of the trial will amount to around 15 / year. Continuing with a one-third recruitment rate this will lead to another 10 over the following two years, leading to an estimated total of 20.

A lower enrolment rate is anticipated within the ‘Midi’ sub-study due to the loss of the joint maternal/infant enrolment within ‘Mama’ and ‘Mini’, as well as the investigations within this sub-study all being additional to clinical care.

## Statistical analysis plan

### Summary of baseline data and flow of patients

Recruitment is pragmatic with an aim to maximise participation from within a limited group. Baseline data and flow of patients will be provided at the final study report.

### Primary endpoint analysis

A descriptive analysis of the endpoints will be completed. For categorical variables (e.g. presence of cataracts) they will be summarised as frequencies (%). For continuous variables (e.g. weight centiles) they will be reported as a mean (standard deviation) or median (inter-quartile ranges) dependent on sample distribution.

For comparisons across CFTRm exposed and non-exposed groups, a chi-square or Fisher’s exact test will be used for categorical variables, and a two-sample t-test or Mann-Whitney U test for continuous variables. Confounders including but not limited to cumulative time of exposure to CFTRm, maternal health, and gestation of offspring will be considered.

In the ‘Mini’ sub-study, enrolling offspring for their first visit up to the age of 12 months allows for maximising sample size although resulting in the possibility of missing early life events. A sensitivity analysis to test assumptions such as only including those up to six months of age will be undertaken.

### Exploratory endpoint analysis

The same approach will be taken as for the primary endpoint analysis.

The interviews in the ‘Mama’ sub-study will be pseudo-anonymised and recorded and transcribed live within MS Teams. The transcripts will be anonymised and imported into NVivo. The interviews will be analysed in this programme using thematic analysis, in a structure similar to that described by Braun and Clarke in their 2006 seminal paper (1). The proposed primary analyser has experience of thematic analysis and grounding in the subject matter. This will be done in an iterative way with analysis happening alongside ongoing data collection. As is common practice in qualitative interviews, the interview guides may be revised if new themes evolve that require further exploration (2,3). To increase the robustness, theme checking will be performed by members of the study team, and member checking will be performed by participants prior to any publication (2,3).

## Interim analysis

With this an observational exploratory study no formal interim analysis will take place.

## Other statistical considerations

Any change in statistical plan will be discussed with an Imperial University statistician and clearly outlined in the final study report.

# Committees in involved in the study

## Study Management Group

A Study Management Group will be formed for this study, comprising grant holders and lead investigators, clinical members of the study team and a study co-ordinator. The role of the group is to monitor all aspects of the conduct and progress of the study, ensure that the protocol is adhered to and take appropriate action to safeguard participants and the quality of the study itself.

## Independent Advisory Group

Co-applicants on the Matriarch_CF grant include lead investigators and senior clinicians from the Mayflowers(10) study group, who have agreed to provide independent review and advice on the design and analysis of this study.

## Patient and Public involvement

A person with CF was also a co-applicant on the Matriarch_CF grant has reviewed the protocol and study design for acceptability and provided input.

Members of the potential study population have also been consulted as part of the study design and will continue to be involved through its duration.

# Direct access to source data

The Investigator/institution will permit study-related monitoring, audits, REC review, and regulatory inspection(s), providing direct access to source data/documents. Study participants are informed of this during the informed consent discussion. Participants will consent to provide access to their medical notes.

# Ethics and regulatory requirements

The Sponsor will ensure that the study protocol, Patient Information Sheet (PIS), Informed Consent Form (ICF) and submitted supporting documents have been approved by a main Research Ethics Committee (REC), prior to any patient recruitment taking place. The protocol and all agreed substantial protocol amendments will be documented and submitted for ethical and regulatory approval prior to implementation.

Before the site can enrol patients into the study, the Principal Investigator must apply for Site Specific Assessment from the Trust Research & Development (R&D) and be granted written confirmation of C&C. It is the responsibility of the Principal Investigator to ensure that all subsequent amendments gain the necessary approval. This does not affect the individual clinician’s responsibility to take immediate action if thought necessary to protect the health and interest of individual patients.

Within 90 days after the end of the study, the CI and Sponsor will ensure that the main REC and HRA are notified that the study has finished. If the study is terminated prematurely, those reports will be made within 15 days after the end of the trial.

# Monitoring plan for the study

The study will be monitored according to the monitoring plan agreed and written by the Sponsor, based on the internal risk assessment procedure. Where appropriate the CI will be asked to complete a copy of the Sponsor’s self-monitoring template. It is the responsibility of the CI to ensure this is completed and submitted to the RO on request (see Study Monitoring Plan). It is the responsibility of the RO to determine the monitoring risk assessment and explain the rationale.

The CI will be provided with a copy of the study monitoring report during the study initiation monitoring visit.

# Finance

This study has been funded by a Strategic Research Centre grant from the Cystic Fibrosis Trust for three years. Additional funding for a paediatric research fellow is covered by Guy’s and St Thomas’ NHS trust and investigator discretionary funds. Funding for a lung physiologist to carry out LCI is also from investigator discretionary funds. Funding was provided by NIHR for a clinician to carry out the interviews in the ‘Mama’ sub-study.

# Insurance and indemnity

NHS bodies are liable for clinical negligence and other negligent harm to individuals covered by their duty of care. NHS Institutions employing researchers are liable for negligent harm caused by the design of studies they initiate. The provision of such indemnity for negligent harm should be stated to the participant.

# Publication policy

Data ownership rights will lie with the institution. Resultant publications or presentations will adhere to Good Publication Practices and International Committee of Medical Journal Editors (ICMJE) guidelines. Results of this study will be submitted as part of MD and doctoral theses and for publication in peer reviewed journals. The CI in conjunction with the clinical research team will prepare the manuscript(s); authorship will be determined by mutual agreement. The CI must review any secondary publications and presentations prepared by investigators.

# Statement of compliance

The study will be conducted in compliance with the protocol, Sponsor’s Standard Operating Procedures (SOPs), GCP and the applicable regulatory requirement(s).

The study conduct shall comply with all relevant laws if directly applicable or of direct effect and all relevant laws and statutes of the UK country in which the study site is located including but not limited to, the Human Rights Act 1998, the Data Protection Act 1998, the Medicines Act 1968, Good Clinical Practice, and with all relevant guidance relating to medicines and clinical studies from time to time in force including, but not limited to the World Medical Association Declaration of Helsinki entitled 'Ethical Principles for Medical Research Involving Human Subjects' (2008 Version), the UK Policy Framework for Health and Social Care Research (as amended).

This study will be conducted in compliance with the protocol approved by the REC and according to GCP standards. No deviation from the protocol will be implemented without the prior review and approval of the Sponsor and REC except where it may be necessary to eliminate an immediate hazard to a research subject. In such case, the deviation will be reported to the Sponsor and REC as soon as possible.

# List of Protocol appendices

**Appendix 1** *Sample Structured Interview Questions*

**Preconception interview**

- Does having CF influence your decision on whether to have a baby, and if so, in what ways?
- If you have been taking Kaftrio, do you think your experience around this medication and its impact influenced your desire for family planning?
- Do you have a feeling for what you’re planning to do about taking Kaftrio during pregnancy? What’s leading you towards this decision?

**Pregnancy interview**

- How has your experience of pregnancy been so far? Any challenges you’d like to share?
- How do you think your CF has impacted on your pregnancy journey? How has your experience been with fitting in your CF therapies?
- What have you been doing with your Kaftrio during pregnancy, and what led you to do this?
- Has your pregnancy been similar or different to your expectations?
- What are your hopes and plans for your delivery, and do you think your CF has influenced your delivery plans?

**Postpartum and early parenthood interview**

- How was your experience of childbirth? Did you feel that your hopes and concerns with regards the birth plan were jointly taken into consideration with regards your cystic fibrosis?
- And how was/is your mental/emotional health in the weeks after delivery?
- Did you have considerations which are CF-specific around breastfeeding? What did you do about your Kaftrio and what led to this decision?
- How do you think your CF impacts on your experience of being a mother?
- And what about your partner’s experience?
- How do you fit in all your health needs around parenthood? (e.g. appointments, physio, medications such as nebulisers?)

**Appendix 2** Limit ages for developmental milestones adapted from Lissauer T, Clayden G. Illustrated textbook of paediatrics. 4th edition. Edinburgh: Elsevier; 2015.

| **Gross motor skills** | | **Vision and fine motor** | | **Hearing, speech, and language** | | **Social behaviour** | |
| --- | --- | --- | --- | --- | --- | --- | --- |
| **Milestone** | **Limit age** | **Milestone** | **Limit age** | **Milestone** | **Limit age** | **Milestone** | **Limit age** |
| *Head control* | 4 months | *Fixes and follows* | 3 months | *Polysyllabic babble* | 7 months | *Smiles* | 8 weeks |
| *Sits unsupported* | 9 months | *Reaches for objects* | 6 months | *Consonant babble* | 10 months | *Fear of strangers* | 10 months |
| *Stands independently* | 12 months | *Transfers objects* | 9 months | *Saying 6 words with meaning* | 18 months | *Feeds self with spoon* | 18 months |
| *Walks independently* | 18 months | *Pincer grasp* | 12 months | *Joins words* | 2 years | *Symbolic play* | 2 – 2.5 years |
|  |  |  |  | *3-word sentences* | 2.5 years | *Interactive play* | 3 – 3.5 years |

# References

1. Jelin AC, Sharshiner R, Caughey AB. Maternal co-morbidities and neonatal outcomes associated with cystic fibrosis ^*^. The Journal of Maternal-Fetal & Neonatal Medicine. 2017 Jan 2;30(1):4–7.

2. CF Foundation. Cystic Fibrosis Foundation Patient Registry 2021 Annual Data Report. 2021.

3. UK Cystic Fibrosis Registry Report 2022. 2022.

4. Taylor-Cousar JL, Emerman I, Odem-Davis K, Vu P, Cameron N, Keller A, et al. WS15.01 Maternal and fetal outcomes in the era of CFTR modulators (MAYFLOWERS) study: interim update. Journal of Cystic Fibrosis. 2024 Jun;23:S27.

5. Taylor-Cousar JL, Jain R. Maternal and fetal outcomes following elexacaftor-tezacaftor-ivacaftor use during pregnancy and lactation. Journal of Cystic Fibrosis. 2021 May;20(3):402–6.

6. Li D, Donnelley M, Parsons D, Habgood MD, Schneider‐Futschik EK. Extent of foetal exposure to maternal elexacaftor/tezacaftor/ivacaftor during pregnancy. Br J Pharmacol. 2024 Aug 21;181(15):2413–28.

7. Collins B, Fortner C, Cotey A, Esther CRJ, Trimble A. Drug exposure to infants born to mothers taking Elexacaftor, Tezacaftor, and Ivacaftor. Journal of Cystic Fibrosis. 2022 Jul;21(4):725–7.

8. Kolaczkowski TJ, Bevan A, Legg J, Self J, Allenby M. Elevated liver function tests in infants exposed to elexacaftor-tezacaftor-ivacaftor in utero and while breastfeeding – Case reports. Journal of Cystic Fibrosis. 2024 Oct;

9. Fortner CN, Seguin JM, Kay DM. Normal pancreatic function and false-negative CF newborn screen in a child born to a mother taking CFTR modulator therapy during pregnancy. Journal of Cystic Fibrosis. 2021 Sep;20(5):835–6.

10. Jain R, Magaret A, Vu PT, VanDalfsen JM, Keller A, Wilson A, et al. Prospectively evaluating maternal and fetal outcomes in the era of CFTR modulators: the MAYFLOWERS observational clinical trial study design. BMJ Open Respir Res. 2022 Jun;9(1):e001289.

11. Vertex Pharmaceuticals. Prescribing Information - Ivacaftor [Internet]. 2012 [cited 2024 Oct 14]. Available from: https://pi.vrtx.com/files/uspi_ivacaftor.pdf

12. Taylor-Cousar JL, Fakhari S, Allison L, Bartels DJ, Jain R, Han S. CF Ferrets exposed to in utero ivacaftor do not develop lens abnormalities. Journal of Cystic Fibrosis. 2024 Sep;

13. Li D, Zhu Y, Donnelley M, Parsons D, Habgood MD, Schneider-Futschik EK. Fetal drug exposure after maternally administered CFTR modulators Elexacaftor/Tezacaftor/Ivacaftor in a rat model. Biomedicine & Pharmacotherapy. 2024 Feb;171:116155.

14. Southern KW, Barben J, Goldring S, Kneen R, Southward S, Rajeev Y, et al. Raised Intracranial Pressure in Three Children with Cystic Fibrosis Receiving Elexacaftor-Tezacaftor-Ivacaftor Modulator Therapy. Am J Respir Crit Care Med. 2023 Jul 1;208(1):103–5.

15. Lhuillier M, Aoust L, Dreano E, Franco-Montoya ML, Landry-Truchon K, Houde N, et al. Elexacaftor/Tezacaftor/Ivacaftor Disrupts Respiratory Tract Development in a Murine Fetal Lung Explant Model. Am J Respir Cell Mol Biol. 2022 Dec;67(6):723–6.

16. Shteinberg M, Lulu A Ben, Downey DG, Blumenfeld Z, Rousset-Jablonski C, Perceval M, et al. Failure to conceive in women with CF is associated with pancreatic insufficiency and advancing age. Journal of Cystic Fibrosis. 2019 Jul;18(4):525–9.

17. Ashcroft A, Chapman S, Mackillop L. The outcome of pregnancy in women with cystic fibrosis: a UK population‐based descriptive study. BJOG. 2020 Dec 16;127(13):1696–703.

18. Thorpe-Beeston JG, Madge S, Gyi K, Hodson M, Bilton D. The outcome of pregnancies in women with cystic fibrosis-single centre experience 1998-2011. BJOG. 2013 Feb;120(3):354–61.

19. Vekaria S, Popowicz N, White SW, Mulrennan S. To be or not to be on CFTR modulators during pregnancy: Risks to be considered. Journal of Cystic Fibrosis. 2020 Mar;19(2):e7–8.

20. Jain R, Kazmerski TM, Taylor-Cousar JL. The modern landscape of fertility, pregnancy, and parenthood in people with cystic fibrosis. Curr Opin Pulm Med. 2023 Nov;29(6):595–602.

21. Jain R, Wolf A, Molad M, Taylor-Cousar J, Esther CR, Shteinberg M. Congenital bilateral cataracts in newborns exposed to elexacaftor-tezacaftor-ivacaftor in utero and while breast feeding. Journal of Cystic Fibrosis. 2022 Nov;21(6):1074–6.

22. Sheeladevi S, Lawrenson JG, Fielder AR, Suttle CM. Global prevalence of childhood cataract: a systematic review. Eye. 2016 Sep 12;30(9):1160–9.

23. Trimble A, McKinzie C, Terrell M, Stringer E, Esther CR. Measured fetal and neonatal exposure to Lumacaftor and Ivacaftor during pregnancy and while breastfeeding. Journal of Cystic Fibrosis. 2018 Nov;17(6):779–82.

24. Bergeron S, Audousset C, Gautier S. P056 Elexacaftor/tezacaftor/ivacaftor and breastfeeding: 3 cases of liver enzymes abnormalities in breastfeed children. Journal of Cystic Fibrosis. 2024 Jun;23:S83–4.

25. Patel P, Yeley J, Brown C, Wesson M, Lesko BG, Slaven JE, et al. Immunoreactive Trypsinogen in Infants Born to Women with Cystic Fibrosis Taking Elexacaftor–Tezacaftor–Ivacaftor. Int J Neonatal Screen. 2023 Feb 21;9(1):10.

26. Braun V, Clarke V. Successful qualitative research, a practical guide for beginners. SAGE Publications; 2013.

27. Lissauer T, Clayden G. Illustrated textbook of paediatrics. 4th edition. Edinburgh: Elsevier; 2015.

28. Short C, Semple T, Saunders C, Hughes D, Irving S, Gardener L, et al. A Short extension to multiple breath washout provides additional signal of distal airway disease in people with CF: A pilot study. J Cyst Fibros. 2022 Jan;21(1):146–54.

29. Saunders C, Jensen R, Robinson PD, Stanojevic S, Klingel M, Short C, et al. Integrating the multiple breath washout test into international multicentre trials. J Cyst Fibros. 2020 Jul;19(4):602–7.
